# Acclimation to different depths by the marine angiosperm *Posidonia oceanica*: transcriptomic and proteomic profiles

**DOI:** 10.3389/fpls.2013.00195

**Published:** 2013-06-17

**Authors:** Emanuela Dattolo, Jenny Gu, Philipp E. Bayer, Silvia Mazzuca, Ilia A. Serra, Antonia Spadafora, Letizia Bernardo, Lucia Natali, Andrea Cavallini, Gabriele Procaccini

**Affiliations:** ^1^Functional and Evolutionary Ecology Lab, Stazione Zoologica Anton DohrnNapoli, Italy; ^2^Evolutionary Bioinformatics Group, Institute for Evolution and Biodiversity, University of MünsterMünster, Germany; ^3^Laboratorio di Proteomica, Dipartimento di Chimica e Tecnologie Chimiche, Università della CalabriaArcavacata di Rende (CS), Italy; ^4^Dipartimento di Scienze Agrarie, Alimentari ed Agro-ambientali, Università di PisaPisa, Italy

**Keywords:** *Posidonia oceanica*, acclimation, ESTs, proteomic, eco-genomic

## Abstract

For seagrasses, seasonal and daily variations in light and temperature represent the mains factors driving their distribution along the bathymetric cline. Changes in these environmental factors, due to climatic and anthropogenic effects, can compromise their survival. In a framework of conservation and restoration, it becomes crucial to improve our knowledge about the physiological plasticity of seagrass species along environmental gradients. Here, we aimed to identify differences in transcriptomic and proteomic profiles, involved in the acclimation along the depth gradient in the seagrass *Posidonia oceanica*, and to improve the available molecular resources in this species, which is an important requisite for the application of eco-genomic approaches. To do that, from plant growing in shallow (−5 m) and deep (−25 m) portions of a single meadow, (i) we generated two reciprocal Expressed Sequences Tags (EST) libraries using a Suppressive Subtractive Hybridization (SSH) approach, to obtain depth/specific transcriptional profiles, and (ii) we identified proteins differentially expressed, using the highly innovative USIS mass spectrometry methodology, coupled with 1D-SDS electrophoresis and labeling free approach. Mass spectra were searched in the open source Global Proteome Machine (GPM) engine against plant databases and with the X!Tandem algorithm against a local database. Transcriptional analysis showed both quantitative and qualitative differences between depths. EST libraries had only the 3% of transcripts in common. A total of 315 peptides belonging to 64 proteins were identified by mass spectrometry. ATP synthase subunits were among the most abundant proteins in both conditions. Both approaches identified genes and proteins in pathways related to energy metabolism, transport and genetic information processing, that appear to be the most involved in depth acclimation in *P. oceanica*. Their putative rules in acclimation to depth were discussed.

## Introduction

The littoral coastal zone is characterized by severe environmental gradients, which mold distribution of populations and species of marine organisms. In a framework of conservation and restoration of biodiversity and in order to predict responses to environmental changes and to develop *ad hoc* conservation strategies, it is crucial to improve our knowledge about the limits of physiological acclimation, physiological plasticity, and intraspecific traits variation, of species living along environmental gradient (Thomas et al., 2004; Schmidt et al., 2008; Thomas, 2010; Hill et al., 2010).

Along the coastline all over the world, excluding polar areas (Green and Short, 2003), seagrasses form among the most productive and neglected marine ecosystems, providing an high number of ecosystem's services, also in comparison to terrestrial habitats (Costanza, 1997; McArthur and Boland, 2006).

Seagrass meadows are very sensitive to disturbance and are being lost rapidly in both developed and developing parts of the world (Short and Wyllie-Echeverria, 1996; Waycott et al., 2009), with only occasional efforts for mitigation and restoration. Seagrass loss has been attributed to a broad spectrum of anthropogenic and natural causes that largely diminish their habitat, affecting their distribution and diversity (Orth et al., 2006; Waycott et al., 2009). For marine plants, seasonal and daily variations in light availability and temperature represent the mains factors driving their distributions along the bathymetric cline. Changes in these environmental factors, due to climatic and anthropogenic effects, can compromise the survival of these key ecosystem-engineering species (Doney et al., 2002).

In Mediterranean Sea, the endemic seagrass *Posidonia oceanica* (L.) Delile can grow as deep as 50 m, depending on light penetration and water clarity (Pasqualini et al., 1998), being extremely sensitive to changes in light availability (Lee et al., 2007). The increase of water turbidity, widely observed as result of human activities along the coastline, affects particularly the deep distribution of the meadows (Ardizzone et al., 2006). *P. oceanica* grows according to a phalanx strategy, with sporadic sexual reproduction and slow-growing clonal lineages, which can persist *in situ* for hundreds of years (Ruggiero et al., 2002; Migliaccio et al., 2005; Arnaud-Haond et al., 2012). Plasticity of *P. oceanica* long-living clones must play an important role on the persistence of the species, being able to survive changes of environmental conditions, as the ones experienced by the unstable highly-impacted Mediterranean coastline.

During the last decades, the application of -*omics* technologies at ecological studies provided powerful tools for following the physiological acclimation in response to environmental variations (Feder and Walser, 2005; Foret et al., 2007; Gracey, 2007; Karr, 2008), and helped researchers to correlate the differences of gene's expression profiles to changes in the main ecological cues in many different organisms (Chevalier et al., 2004; Edge et al., 2008; Kassahn et al., 2009; Larsen et al., 2012; Richards et al., 2012).

Despite their high ecological value, seagrasses are poorly understood for what concerns the genetic basis behind their physiological adaptation and plasticity (Procaccini et al., 2007). It's only recently that transcriptomic approaches were implemented for few species, to correlate seagrasses gene expression with ecological factors. In particular, transcriptomic response to temperature changes and thermal stress was studies in the two congeneric species, *Zostera marina* and *Zostera noltii* (Maathuis et al., 2003; Reusch et al., 2008; Massa et al., 2011; Winters et al., 2011), while transcriptional (Bruno et al., 2010; Serra et al., 2012b) and proteomic approaches (Mazzuca et al., 2009) were applied to study light response in natural conditions in *Posidonia oceanica*. In *P. oceanica*, studies were hampered by the fact that available genomic and transcriptomic resources only consisted in a single Expressed Sequences Tags (EST) library, obtained from shoots collected along a depth range (from −5 to −30 m) in a single site (Wissler et al., 2009), and available in Dr.Zompo, a specific seagrasses database containing both *P. oceanica* and *Z. marina* EST sequences http://drzompo.uni-muenster.de/ (Wissler et al., 2009).

Several approaches can be utilized for genomic studies in species for which the whole genome is not available (e.g., Hofmann et al., 2005; Stapley et al., 2010), most of them requiring high computational power and advanced bioinformatics resources (Morozova and Marra, 2008; Pop and Salzberg, 2008; Metzker, 2010). Among the others, Suppressive Subtractive Hybridization (SSH)–EST library (Diatchenko et al., 1996) approach resulted especially powerful to identify differentially expressed genes in the presence of clear differences in physiological status (Jones et al., 2006; Puthoff and Smigocki, 2007) and it was applied to study flowering (Matsumoto, 2006), senescence (Liu et al., 2008a,b), or salt-stress (Zouari et al., 2007) in terrestrial plants.

The aim of this work was to identify differences in transcriptional and proteomic profiles in *P. oceanica*, correlated with its bathymetric distribution, with the ultimate goal to identify the metabolic pathways involved in acclimation. We also aimed to increase genomic resources in *P. oceanica* and to present a powerful approach for studying physiological response at a molecular level in organisms for which genomic resources are limited.

In order to do that, we built a SSH-library between plants growing at two different depths in the same meadow, and we obtained their protein content using the innovative USIS mass spectrometry methodology coupled with 1D-SDS electrophoresis. Proteins identifications were performed using the Global Proteome Machine (GPM) open-source system for analyzing, storing, and validating proteomics information derived from tandem mass spectrometry (Craig et al., 2004; Fenyö et al., 2010) and X!Tandem software (Craig and Beavis, 2003; Craig et al., 2005) against a local database derived by Dr.Zompo and UniProtKB databases.

## Materials and methods

### Shoots sampling

*Posidonia oceanica* shoots were collected by SCUBA diving in the Lacco Ameno meadow, Island of Ischia (Gulf of Naples, 40°45′52′′ N; 13°53′29′′ E) at two sampling stations located above and below the summer thermocline (−5 and −25 m depths).

Leaf tissue from 20 shoots for each stand was cleaned from epiphytes and shock frozen in dry ice on the research vessel soon after collection. Tissue was stored at −80°C before RNA and proteins extraction.

Temperature, salinity and Photosynthetic Active Radiation (PAR) were measured at the surface and at six different depths along the bathymetric distribution of the meadow (Table 1). Values were obtained right before shoot sampling, using a Seabird Seacat Probe operated from the boat and connected to a wired computer onboard.

**Table 1:** **Environmental variables**.

**Depth (m)**	**Temperature (°C)**	**Salinity (PSU)**	***PAR* (μM/m^2^/sec)**
0	27.55	37.79	960
**−5**	**26.84**	**37.78**	**703**
−10	24.03	37.75	491
−15	22.24	37.74	355
−20	20.02	37.78	230
**−25**	**18.99**	**37.78**	**100**
−30	18.12	37.79	50

Values of temperature, T (C°); salinity (PSU); and Photosynthetically active radiation, PAR (μM/m^2^/sec) collected during the sampling (July, 2010 h ~14:00) with a Seabird Seacat Probe at the surface and at six depths along the bathymetric distribution of the meadow (depth). Sampling stations are indicated in bold.

### RNA extraction

Total RNA was isolated from leaf tissue of ten shoots for each condition, using hexadecyltrimertihyl ammonium bromide (CTAB) method (Chang et al., 1993) with some modifications. About 4 g of each shoot were weighted and grind to a fine powder in liquid nitrogen in a pre-cooled mortal. The powder was transferred to an Eppendorf tube and 1 ml of pre-warmed extraction buffer was added to the samples (2% CTAB, 0.2% β-mercaptoethanol, 1.4 M NaCl, 20 mM EDTA, 200 mM Tris-HCl pH 7.5). After incubation at 65°C for 10 min, 800 μl chloroform-isoamyl alcohol (49:1 v/v) were added. After centrifugation, at 6500 rpm for 10 min, the RNA was selectively precipitated from the upper phase through the addition of 1/4 volume 10 M LiCl and precipitated for 2–4 h at −20°C. RNA was recovered by centrifugation (Beckman JA-20 rotor) at 11,000 rpm at 4°C for 30 min. Supernatant containing genomic DNA was removed and pellets were washed once with 1 ml 100% EtOH and two times with 1 ml 75% EtOH. Precipitations were followed by centrifuging at 10,000 rpm for 5 min to remove the EtOH and pellets were dried at room temperature for few minutes. RNA was suspended in 50 μ l H_2_O RNase free. RNA quality and quantity was evaluated by gel electrophoresis and by Nano-Drop (ND-1000 UV-Vis spectrophotometer; NanoDrop Technologies) monitoring the absorbance at 260 nm. Purity was determined by 260/280 nm and 260/230 nm ratios using the same instrument. All samples resulted free from protein and organic solvents used during RNA extraction. RNA was stored at −80°C.

### Construction of suppressive subtractive hybridization (SSH)-libraries

For each depths considered in the experiment, the same quantity of total RNA extracted from individual shoot was pooled. About 280 μg of each RNA pools were purified using Dynabeads mRNA Purification kit (DYNAL BIOTECH), following the manufactures instructions, in order to select polyA^+^ mRNA.

The construction of the forward and reverse SSH libraries was performed using the PCR-select cDNA subtraction kit (Clontech, Palo Alto, CA, USA), following the manufacturers instruction. Shallow library (FORWARD subtraction, S) was carried out with shallow mRNA as tester pool and deep mRNA as driver pool. Reversely, in the deep library (REVERSE subtraction, D), deep mRNA was used as tester pool and shallow mRNA as driver pool.

The two resulting subtractive libraries were cloned individually in pCR2.1-TOPO vector (Invitrogen), and transferred into TOP F' cells (Invitrogen) with vector: insert ratio 1:10, following manufacturer's instructions. Colonies were grown overnight in Petri dishes with LB medium and Ampicillin (μg/ml). Afterwards, single colonies were picked and transferred into 96-well plates containing LB and Ampicillin (LB/Amp) to grow overnight. About twenty 96-well plates for each library (S and D) were screened in PCR to identified positive recombinant colonies. Every single colony has been amplified using specific primers of the TOPO vector: T7 forward and M13 reverse. PCR products have been analyzed on 1.5% Agarose gel stained with Ethidium Bromide in 1× TAE buffer. For each library, about 1000 colonies having an insert longer than 500 bp were selected for sequencing (data not shown).

Finally, replicates of selected colonies were stored in LB/Amp-15% glycerol (at −80°C) and shipped to the Biologisch-Technische Produkte Service of the Max Planck Institute for Molecular Genetics (Molgen, Berlin, DE) for ESTs sequencing using ABI 3730xl automated DNA sequencers (Applied Biosystems, USA).

### Data analyses and bioinformatics

Bioinformatics analysis of EST data sequences was carried out by the Evolutionary Bioinformatics Group at the Westfälische Wilhelms University Institute for Evolution and Biodiversity (Münster, DE).

Raw sequences of each library were trimmed removing the low quality regions, the vector, the adapter and the poly-A/T regions, using PREGAP4 (Staden, 1996). Only the EST raw sequences longer than 100 nucleotides entered the assembly step. Successfully trimmed EST reads were assembled into tentative unigenes (TUGs) using CAP3 (Huang, 1999). After trimming and deletion of short sequences (94 in total), only sequences of good quality were finally assembled into 486 TUG, which include 2290 ESTs. Considering other 286 single reads (Singletons), a total of 772 SSH-Unigenes were identified. To infer functions of SSH-Unigenes, an homology search, using BLASTN algorithm, was made against public multiple databases: non-redundant NCBI Gene Ontology (GO), KEGG (Kyoto Encyclopedia of Genes and Genomes), SWISSPROT, and NR-NCBI (using BLASTX algorithm with an Expect-value threshold of = 0.001) and Dr.Zompo. Identified Unigenes were stored in the database Dr.Zompo as “Pooc_B” library. Divergence in gene expression patterns at the two different depths, was assessed mapping Unigenes into functional categories using Mapman (Thimm et al., 2004).

### Protein extraction and electrophoresis

Only adult leaves were used for this purpose according to Spadafora et al. (2008). Plant material was grounded to a fine powder in liquid N_2_ using mortar and pestle and transferred to a centrifuge tube, where cold 10% trichloroacetic acid in acetone with 0.1 M β-mercaptoethanol, was added. Samples were kept at −20°C for at least 2 h, and then centrifuged at 12,000 g for 15 min at 4°C. The resulting pellet was washed 3 times by suspending in acetone containing 0.1 M DTT and centrifuged as above between each wash. The pellet was air-dried and used for protein extraction. Tissue powder from ten different plants from shallow (A) and deep (B) conditions, respectively, was pooled and used for phenol-based protein extraction (Spadafora et al., 2008). Tissue powder was re-suspended in extraction buffer containing 0.1 M Tris-HCl, pH 8.8, 2% SDS and 0.1 M β-mercaptoethanol. Supernatant was mixed with equal volume of buffered phenol (pH 8.0, Sigma). Phases were separated by centrifugation at 15,000 g for 5 min. The phenol phase was precipitated with 5 volumes of cold methanol containing 0.1 M ammonium acetate overnight (−20°C). Protein phase was recovered by centrifugation and washed twice with cold acetone. The phenol extraction step has been repeated twice for each set of samples and thereafter processed for mass spectrometry analyses.

**Figure 1:**
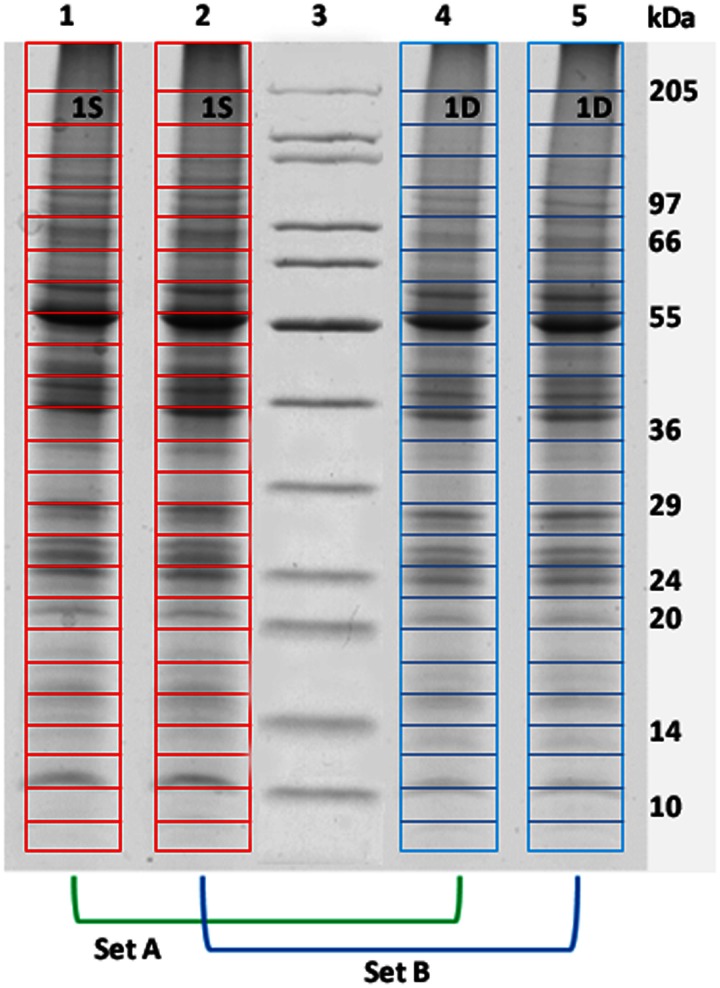
**One-dimensional-SDS-PAGE.** One-dimensional-SDS-PAGE of two independent biological replicates of leaf proteins purified from shallow (lanes 1 and 2) and deep (lanes 4 and 5) *P. oceanica* plants. Each gel lane was cut in 24 slices, then each couple (1S;1D…24S;24D) from swallow and deep lanes was comparatively analyzed by mass spectrometry as Sets A and B datasets.

Protein samples from Sets A and B were processed on 1D SDS-PAGE; the Laemmli buffer system was used to cast a 6% stacking gel and 12.5% resolving gel. After denaturation at 100°C for 3 min, proteins were resolved at constant voltage (200 V) in a Bio-Rad mini Protean II apparatus. CBB stained gels were scanned on a densitometer (GS800, Biorad) and peptide bands were quantified using QuantityOne software (Bio-Rad). 1D gel lines from Sets A and B samples were cut in 24 slices each (Figure 1) and digested enzymatically with trypsin. The tryptic fragments were analyzed by LC-ESI MS/MS coupled with the ionization source for mass spectrometers named Universal Soft Ionization Source (USIS) (Cristoni S, patent no. PCT/EP2007/004094). For the experiments, a Bruker HTC Ultra spectrometer, equipped with a Dionex Ultimate 3000 HPLC system, was used. Chromatography separations were conducted on a Thermo Biobasic C18 column (1 mm i.d. _ 100 mm length and 5 μm particle size), using a linear gradient from 5 to 90% acetonitrile (ACN), containing 0.1% formic acid with a flow of 100 μ L/min, including the regeneration step; one run lasted 70 min. Acquisitions were performed in the data-dependent MS/MS scanning mode (full MS scan range of m/z 250–2000 followed by full MS/MS scan for the most intense ion from the MS scan).

### Protein identification

Spectra acquired by LC-MS/MS were used to identify peptide sequences using the open-source system GPM software against the GPM plant database (http://plant.thegpm.org/tandem/thegpm_tandem.html). Since the GPM plant database considers only a few species belonging to *Liliopsida*, excluding seagrasses, this procedure can lead to a loss of peptide identification by mass spectrometry. Thus, spectra acquired by LC-MS/MS were also used to identify peptide sequences using X!Tandem software (http://www.thegpm.org/tandem/index.html) against a local database. X!Tandem is a search engine for identifying proteins by searching sequence collections, reducing the time required to match protein sequences with tandem mass spectra (Craig and Beavis, 2003). It scores the match between an observed tandem mass spectrum and a peptide sequence, by calculating a score that is based on the intensities of the fragment ions and the number of matching b- and y-ions. This score is converted to an expectation value using the distribution of scores of randomly matching peptides (Fenyö et al., 2010).

In the local database, sequences from seagrasses and other species belonging to *Liliopsida* available in the UniprotKB database and the amino acid sequences of *P. oceanica* and *Z. marina* deduced from five ESTs libraries (Pooc_A, Pooc_B, Zoma_A, Zoma_B and Zoma_C) collected in the Dr.Zompo database (Wissler et al., 2009, http://drzompo.uni-muenster.de/) were included. In the last case, it has been necessary first to create a protein database from the nucleotide sequences. For this, the most straightforward procedure is listing all possible ORFs from the six reading frames; the resulting list contains a large majority of protein sequences that are unlikely to be real, but MS/MS data allow to discriminate between real and false polypeptide sequences (Armengaud, 2009). The use of all possible reading frames has allowed to optimize the peptide identifications. ESTs are relatively error prone (Alba et al., 2004) and an ORF can be split and displayed over 2 or 3 frames when a frame-shift error exists on the cDNA sequence. Consequently, the deduced protein sequence can be incorrect (Serra et al., 2012a). The translation of each nucleotide sequence was performed using a translation tool available at http://www.ebi.ac.uk/Tools/st/emboss_transeq/5.

## Results

### SSH-library

After assembly process and trimming, ESTs sequences clustered to 772 TUGs, 286 of which were Singletons and 486 were Contigs, consisting of two or more reads. Among the TUGs identified, the 45% (349/772) had a GO annotation, while the 55% (423/772) were not classified in GO. Protein annotation against SwissProt database, gave in total 278 Unigenes classified into putative known functions or unclassified proteins. Based on Dr.Zompo database, only the 39% of the total number of SSH-Contigs (189/486) had homologies with known *P. oceanica* ESTs sequences, while 61% (297/486) were new. The main statistic features of the SSH–EST library are reported in Table 2, other data are reported in Table S1.

**Table 2:** **EST library features**.

	***N*°**
ESTs in shallow library	1330
ESTs in deep library	1246
Contigs in shallow library	200
Contigs in deep library	314
Contigs in common (shallow + deep)	28
Singletons only in shallow library	139
Singletons only in deep library	147

Comparison of main statistical features between shallow and deep Posidonia oceanica EST libraries.

Annotation and other features of SSH-TUGs are listed in Tables S2a,b. TUGs were included in the database Dr.Zompo (http://drzompo.uni-muenster.de/) in the *P. oceanica* “Pooc_B” library. The 2576 single ESTs obtained were submitted to the dbEST within GeneBank (LIB EST_Pooc SSH, Genbank Accession Numbers: JZ354020–JZ356595).

### Comparison of tentative unigenes frequencies between shallow and deep conditions

Among the 486 Contigs identified, only 28 (3% of the total) were present in both libraries, while 314 Contigs have been found only in the deep-library (D-library) and 200 Contigs only in the shallow-library (S-library). For Singletons, 147 and 139 were present only in the D-library and in the S-library, respectively (Table 2).

TUGs more abundant in the S-library include (i) proteins involved in protein turnover, as Proteasome subunit alpha, E3 ubiquitin (F-box protein) and ATP-dependent Clp protease proteolytic subunit and (ii) proteins involved in stress defense, as Heat shock cognate 70 kDa protein, Ketol-acid reductoisomerase, Acyl-CoA-binding protein and Cytochromes c/b subunits (Table S2a). TUGs more abundant in the D-library include (i) proteins involved in the photosynthetic pathways as Chlorophyll a-b-binding proteins, Photosystem I/II, Oxygen-evolving enhancer protein and (ii) proteins involved in basal metabolism and in stress response, as Universal stress protein, Zinc-finger protein, Metallothionein-like protein, Cytochrome P450, Caffeoyl-CoA O-methyltransferase, Aquaporin PIP2 and S-norcoclaurine synthase (Table S2b).

Among Contigs, only six showed significant differences in frequency (*p* ≤ 0.05) between libraries. Five Contigs were up-regulated in S-library and only one was up-regulated in D-library (Table 3). The differential expression of two of these Contigs, Pooc_B_c42, encoding for a N(2),N(2)-dimethylguanosine tRNA methyltransferase, and Pooc_B_c217, whose function is unknown, has been tested in RT-qPCR and showed the expected expression profiles (Figure S1, also see Serra et al., 2012b).

**Table 3:** **List of Contigs differentially expressed**.

**Contig**	**Annotation**	**Best hit**	***E*-value**	**Shallow library (−5 m)**	**Deep library (−25 m)**	**Regulation**	**RT-qPCR regulation**
**Pooc_B_c42**	N(2),N(2)-dimethylguanosine tRNA methyltransferase	Q34941	3.0e-14	15	0	*UP-5 m*	*UP-5 m*
Pooc_B_c444	F-box protein At5g67140	Q9FH99	2.0e-28	16	0	*UP-5 m*	
Pooc_B_c209	no hit	–	–	15	0	*UP-5 m*	
Pooc_B_c205	no hit	–	–	341	19	*UP-5 m*	
Pooc_B_c18	no hit	–	–	38	0	*UP-5 m*	
**Pooc_B_c217**	cellular_component	GO:0005575	–	0	17	*UP-25 m*	*UP-25 m*

*List of Contigs showing significant EST frequency difference between libraries (p ≤ 0.05). For each Contig, name, annotation, best hits E-value, number of EST reads in each library, and regulation signals are indicated. Differential expression between the two depths reported in this work (shallow −5 m, deep −25 m) was tested in RT-qPCR experiment by Serra et al. (2012b). RT-qPCR results are also reported in Figure S1 for Contigs Pooc_B_c42 and Pooc_B_c217 (in bold)*.

Since SSH technique can also generate background clones which are not representing differentially expressed sequences but can be false positives, we will consider the remaining transcripts identified here as “putative differentially expressed” until each one will be experimentally validated in future studies.

**Figure 2:**
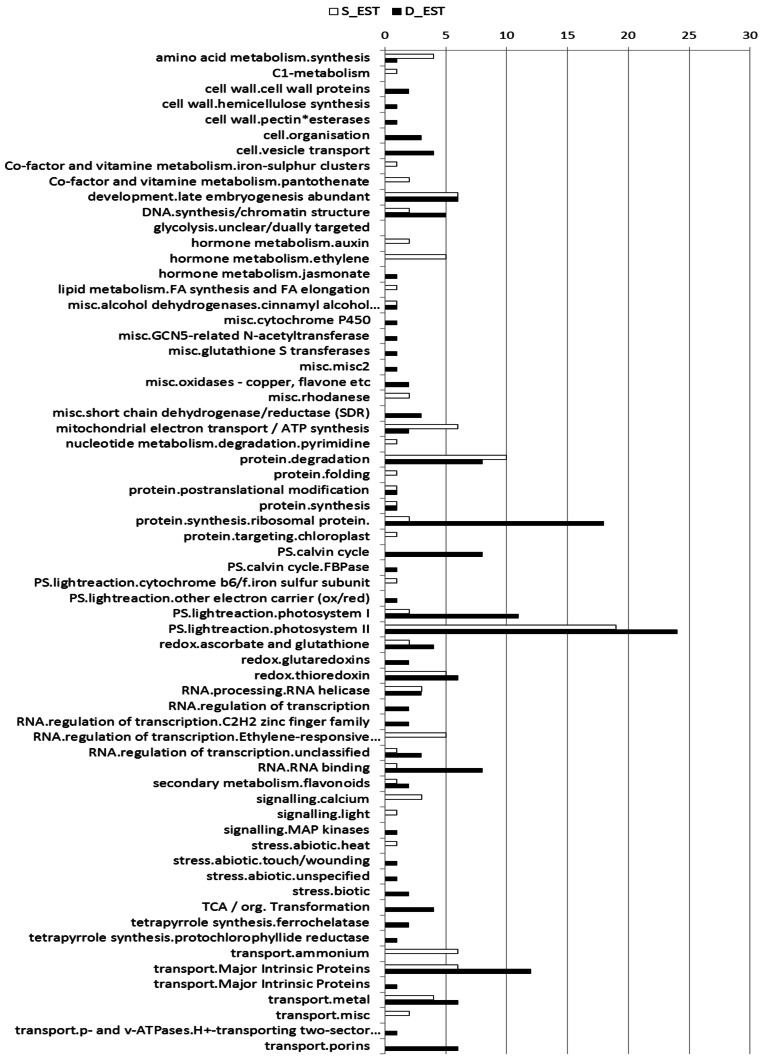
**Expression Level of TUGs.** Level Expression (number of EST) of TUGs associated to the different metabolic categories in shallow (white column) and deep (black column) conditions.

Differences between libraries were both quantitative, i.e., relative expression of particular Unigenes, assessed as number of reads, and qualitative, i.e., comparing proteins for the same functional categories or the same metabolic pathways. The comparative abundance of each functional category is shown in Figure 2. Genes belonging to light related processes (e.g., photosynthesis and energetic metabolism), genetic information processing (e.g., transcription and translation), transport, folding, sorting, and degradation of proteins were abundant in both conditions. Nevertheless, looking at different pathways, differences were observed in their protein composition. For the photosynthetic pathway, in the D-library there are 26 different TUGs encoding for Chlorophyll a-b-binding proteins, whereas only 15 different TUGs were present in the shallow one (Tables S2a,b). The opposite trend was observed for proteins related to electrons carrier transport (Tables S2a,b). TUGs assigned to PSI and PSII were more abundant in low light (D-library) rather than in high light (S-library), and this difference was particularly strong for PSI (PSI: 2/19, PSII 19/24 reads in S- and D-library, respectively, Figure 2). Other striking qualitative differences were observed among stress response proteins. Universal stress proteins, as Zinc-finger, Metallothionein-like, Cytochrome P450, Caffeoyl-CoA O-methyltransferase, Aquaporin PIP2 and S-norcoclaurine synthase were more abundant in the D-library (Table S2b), while other proteins involved in stress defense, Heat shock cognate 70 kDa, Ketol-acid reductoisomerase, and Acyl-CoA-binding protein were more abundant in the S-library. TUGs belonging to protein turnover, such as proteasome subunit alpha, E3 ubiquitin (F-box protein), ATP-dependent Clp protease proteolytic subunit (Table S2a) were more abundant in the S-library.

**Figure 3:**
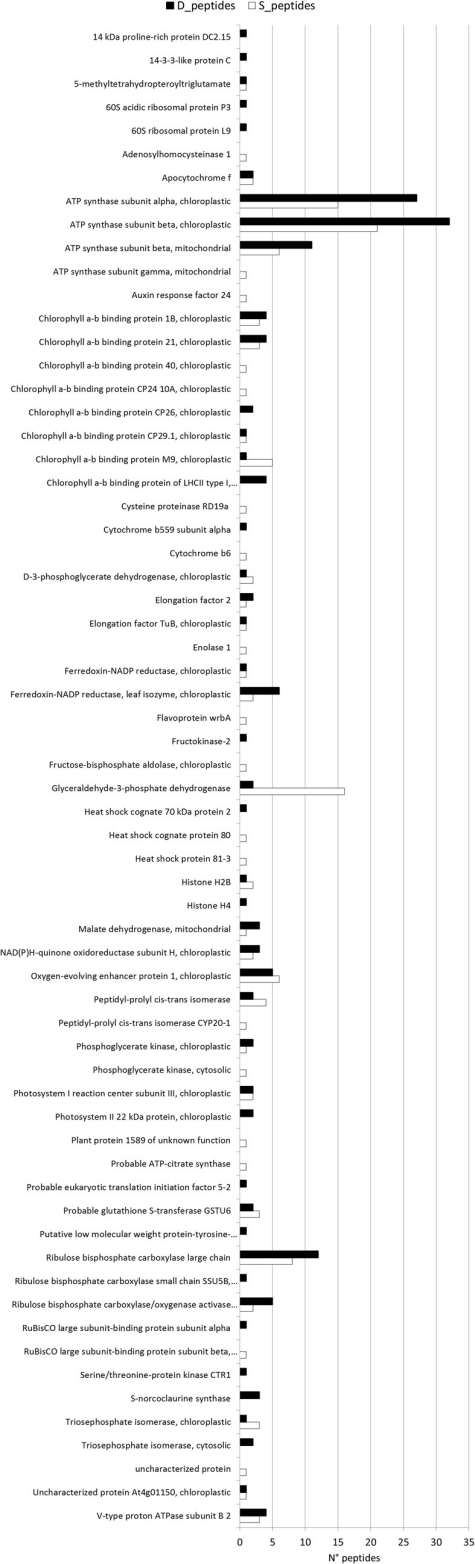
**Expression Level of peptides.** Level of expression (number of peptides) associated to the different proteins in shallow, (white column) and deep (black column) conditions.

**Figure 4:**
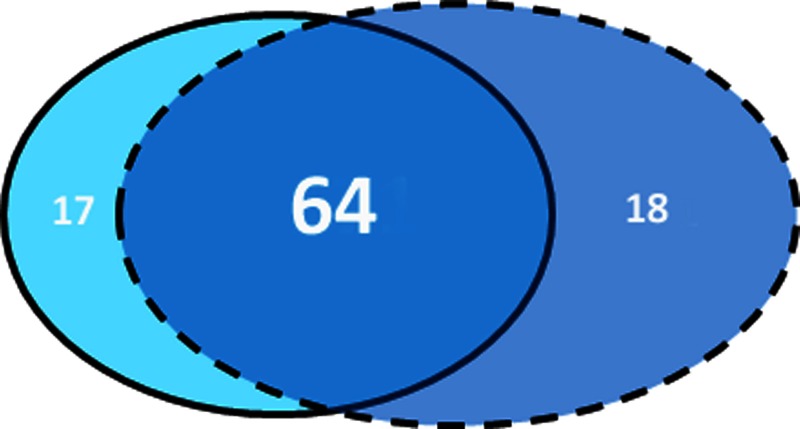
**Venn diagrams.** Venn diagrams comparing the total protein discovery with those found only in shallow or in deep samples. Among the 64 newly identified proteins, 17 are exclusive of shallow samples and 18 of the deep ones.

Peptide sequences from shallow and deep samples, their relative positive match against the different databases and their functional annotations are reported as in Tables S3a (shallow) and S3b (deep). Database search methods using the GPM and X!Tandem software combined with classical BLASTN searching method to identify peptide sequences, have allowed to assign the large portion of the identified peptides at proteins with known function, enhancing significantly our previous knowledge on the *P. oceanica* proteome. After eliminating redundancies (i.e., proteins common to the two sets of data), the total net protein discovery amounts exactly to 64 proteins, which were principally involved in photosynthesis and energy metabolism, with both structural and regulative functions (Figure 3). Mitochondrial and chloroplastic ATP synthase subunits were the most abundant. The chloroplast isoforms of ATP synthase, which take part to the Calvin cycle, were highly expressed in both light conditions, while the mitochondrial isoforms, which take part to respiration, appeared down regulated in low light. Proteins involved in photosynthetic metabolic pathways, such as oxygen-evolving enhancer proteins, were almost equally represented in the two conditions. Though most of the identified proteins showed little differences in number of peptides between the two conditions, 17 unique peptides were found only in shallow samples corresponding to as many proteins (Table 4A); meanwhile, in deep samples, 23 unique peptides that were not found in shallow ones have been assigned to 18 proteins (Table 4B). Summarizing the results, among the 64 newly identified proteins, 17 are exclusive of shallow samples and 18 of the deep ones as shown by the Venn diagrams (Figure 4). RuBisCO subunits, Chlorophyll a-b-binding proteins and Ferredoxin-NADP reductase (leaf isozyme) were more represented in the deep samples, while Glyceraldehyde-3-phosphate dehydrogenase was more represented in the shallow ones. Moreover, proteins with regulative activity, as the Ribulose bisphosphate carboxylase/oxygenase activase A and a 14-3-3-like protein, were also recognized as more expressed in shallow in respect to the deep samples.

**Table 4:** **List of unique peptides**.

**Sample slice**	**Protein attribution**	**Log(e) peptide**	**Peptide sequences**	**TBLASTN (Drzompo)**	***E*-value (Drzompo)**	**Functional annotation**	**Mr (kDa)**
**(A)**							
4S	gi|38154488| gb|AY368906|-1 gpmDB [20/28] protein	3.3	MAEAETFAFQAEINQLLSLIINTFYSNK	Zoma_C_c66842	2.00e-08	Heat shock protein 81-3	80.1
4S	gi|38154492| gb|AY368907|-1 gpmDB [21/29] homo (3/3) protein	−6.7	DLVLLLFETALLTSGFSLEEPNTFGNR	Pooc_Contig353	1.00e-10	Heat shock cognate protein 80	80.1
7S	At3g23810.1 gpmDB [83/146] homo (10/10) protein	−4.2	WVFPDTNSGIIVLAEGR	Zoma_C_c68954	0.001	Adenosylhomocysteinase 1	53.1
7S	gi|119350|sp|P25696.1	−2.8	SGETEDTFIADLAVGLSTGQIK	Zoma_C_c67987	3	Enolase 1	47.9
7S	At2g28000.1 gpmDB [61/93] homo (5/5) protein	−10	GGYPILIIAEDIEQEALATLVVNK	Zoma_C_c58171	2	RuBisCO large subunit-binding protein subunit beta, chloroplastic	62.9
8S	ATCG00490.1 gpmDB [74/111] homo (0/33)protein	−56.9	TFQGPPHGIQVER	Pooc_B_rp6_D3_R	2.7	Auxin response factor 24	50.5
10S	gi|48752579| gb|CO083098|-3 gpmDB [10/11] homo (1/1) protein	−3.1	TLLVSAPGLGDYISGAILFEETLYQSTIDGK	Pooc_PC035C04	3.00e-00	Fructose-bisphosphate aldolase, chloroplastic	32.7
10S	gi|156725011| gb|EV229122|-1 gpmDB [1/7] homo (2/93) protein	−30.2	FGIVEGLMTTVHSITATQK	Pooc_Contig14	6e-05	Glyceraldehyde-3-phosphate dehydrogenase	54.7
10S	gi|82621107| gb|DQ284454|-3 gpmDB [8/10] homo (0/3) protein	−8.6	NDLEFAKKLASLADLYVNDAFGTAHR	Pooc_PC021E08	2.00e+00	Phosphoglycerate kinase, cytosolic	52.1
10S	gi|150162092| gb|EE553762|-1 gpmDB [0/1] protein	−2.4	DALFKHANIKPIITSTVWK	Pooc_PC044A11	0.22	Plant protein 1589 of unknown function	16.8
12S	gi|73880486| gb|DT483224|3 gpmDB [0/1] homo (5/5) protein	−1.9	NPLNYTQVSVLADDILK	Zoma_C_c45955	0.004	ATP synthase subunit gamma, mitochondrial	32.7
15S	Pooc_B_rp7_C5_R_6	−12.7	NSPNSFDPLGLAEDPEAFAELK	Pooc_B_rp7_C5_R	2.00e-08	Chlorophyll a-b-binding protein 40, chloroplastic	31.6
16S	gi|52390306| gb|CV233595|3 gpmDB [0/1] homo (12/12)	−2.7	LTGTDVGYPGGLWFDPLGWGSGSPEK	Pooc_B_c320	0.28	Chlorophyll a-b-binding protein CP24 10A, chloroplastic	26.6
16S	gi|48389884| gb|CN917384|-3 gpmDB [9/10] protein	−2.9	NEVPVISPEQLAEADGIIFGFPTR	Zoma_C_c56810	2	Flavoprotein wrbA	22.7
17S	gi|38605705|sp|P05642.2|	−3.1	IVTGVPEAIPVIGSPLVELLR	Zoma_C_c33944	9	Cytochrome b6	33.4
17S	gi|73875282| gb|DT478020|-3 gpmDB [0/1] homo (2/2) protein	−6	IVIGLFGDDVPQTAENFR	Pooc_PC015E05	8	Peptidyl-prolyl cis-trans isomerase CYP20-1	31.3
**(B)**							
4D	sp|Q6L509|Q6L509_ORYSA	−11.2	IINEPTAAAIAYGLDK	Zoma_C_c66491	0.005	Heat shock cognate 70 kDa protein 2	70
6D	gi|116010686| gb|AK241321|-2	−1.8	GGECVGGGGGGGGGGGAEAR	Pooc_Contig164	0.0000001	60S ribosomal protein L9	88
6D	gi|194694909|gb|BT036534|-3	−1.8	GDVADGVFLGHADWPR	Pooc_B_c93	9.6	Probable eukaryotic translation initiation factor 5-2	51.6
7D	At2g28000.1 gpmDB [72/113] homo (6/19) protein	−9.3	APLLIIAEDVTGEALATLVVNK	Zoma_C_c57597	0.00003	RuBisCO large subunit-binding protein subunit alpha	52.3
10D	gi|187950292| gb|AY103880|-3 gpmDB [0/1] protein	−4.8	LVDTNGAGDAFVGGFLSQLVLGK	Pooc_PC010D04	0.87	Fructokinase-2	55.5
12D	gi|34959481| gb|CA106174|-2 gpmDB [0/1] protein	−1.7	AARPPPAGTPPPR	Pooc_PC019C02	0.26	Putative low molecular weight protein-tyrosine-phosphatase slr0328	28.5
13D	At5g65430.1gpmDB [53/81]homo (38/38) protein	−3.7	QAFEEAIAELDTLGEESYK	Pooc_PC039D11	0.00001	14-3-3-like protein C	28
14D	At4g10340.1gpmDB [40/73]homo (12/12) protein	−4.1	TGALLLDGNTLNYFGK	Pooc_Contig159	0.00005	Chlorophyll a-b-binding protein CP26, chloroplastic	30.1
14D	gi|110373880|gb|EC938302|3 gpmDB [0/3] homo (0/7) protein	−14	QEDIDGFLVGGASLK	Zoma_C_c59451	0.028	Triosephosphate isomerase, chloroplastic	34.5
15D	gi|45990591| gb|CN149099|-3gpmDB [0/5]homo (9/51) protein	−12	TDEFPGDYGWDTAGLSADPETFAK	Pooc_B_c360	1.00e-09	Chlorophyll a-b-binding protein of LHCII type I	33
15D	gi|73873524| gb|DT476262|2 gpmDB [0/6] homo (0/14) protein	−10.8	SEIPEYLTGEVPGDYGYDPFGLSK		0.00004		
15D	Zoma_C_c15686_5	−10	ELEVIHTRWAMLGTLGCVFPELLSR		0.0006		
15D	gi|157980300| gb|EX528572|-3 gpmDB [0/12] homo (0/15) protein	−29.5	YLGSFSGEAPSYLTGEFPGDYGWD TAGLSADPETFAK		1.00e-15		
15D	Zoma_C_c34383_5	−10.7	EPNSIFGVGGITMRRNTVK	Pooc_B_c132	0.005	Chlorophyll a-b-binding protein 21, chloroplastic	28.2
16D	Pooc_Contig333_3	−21.8	SKVEDGIFGTSGGIGFTK	Pooc_Contig333	0.00003	Photosystem II 22 kDa protein, chloroplastic	29.3
16D		−10.3	VAMLGFAASIFGEAITGK		0.00003		
18D	Pooc_PC011B10	−18	TEIEGDGGVGTTTK	Pooc_PC011B10	0.001	14 kDa proline-rich protein DC2.15	14.3
18D	Pooc_Contig48_2	−11.5	VWDFCGSSQLMQLLPK	Pooc_Contig48	0.000002	S-norcoclaurine synthase	23.3
		−10.2	QIEGGHLDLGFLSSHSR		0.000007		
	Pooc_Contig132_3	−18	VWDFCASCQLMQLLPK	Pooc_Contig132	7.00e-07		
21D	At5g38430.1 gpmDB [31/51] homo (6/6) protein	−3	EHGNTPGYYDGR	Pooc_Contig3	0.001	Ribulose bisphosphate carboxylase small chain SSU5B, chloroplastic	18.9
22D	LOC_Os01g61920.1 homo (267/267)	−2.5	TVTAMDVVYALK	Pooc_B_rp10_E10_R	0.018	Histone H4	11.4
23D	gi|27548338| gb|CA766549|	−3.3	FDSLEQLDEFSR	Zoma_C_c60643	1.6	Cytochrome b559 subunit alpha	9.3

Log (e): the base-10 log of the expectation that any particular peptide assignment was made at random.

List of unique peptides identified within the 23 slices of 1DE gel of proteins from (A) shallow samples and (B) deep samples. In the table are shown: the protein attribution obtained by GPM and X!TANDEM softwares with the corresponding log(e) values, the peptide sequence, the annotation obtained with TBLASTN search against Dr.Zompo database, with the corresponding E-values and putative functions, the molecular mass of the protein sequence, in kilodaltons.

## Discussion

The aim of this work was to investigate physiological acclimation in *Posidonia oceanica* plants along a bathymetric gradient, combining transcriptomic, and proteomic analyses. Plants were collected after the stabilization of the summer thermocline, when light and temperature regimes were well-differentiated between the two selected sampling stations (−5 and −25 m).

A not perfect match between transcriptome and proteome profiles was found, since some targets were identified only in one of the two datasets (e.g., F-box protein only among ESTs; RuBisCO only among peptides). Despite the correlation between transcriptomic and proteomic profiles is usually high (e.g., Guo et al., 2008), the effective level of observed accordance among these data varies in dependence of the system studied (Pascal et al., 2008; Vogel and Marcotte, 2012).

Furthermore, as also reminded from other authors (Feder and Walser, 2005; Diz et al., 2012) in interpreting differences in transcripts and peptides abundance, it should be taken in to account that many different regulative steps during transcription and translation processes influence the expression levels of mRNAs and their corresponding protein.

Besides the problem of reading correctly differences in genes and proteins expression profiles, a main question in combining transcriptional and proteomic data analyses is also in how to asses the interaction between them (Rogers et al., 2008; Huang et al., 2013). In the present work, data obtained from both analyses were discussed jointly, in order to asses the putative role and function of each target recognized in *P. oceanica* acclimation to depth.

Overall, our results suggested that a large portion of genes and proteins which were identified as *putatively* differentially expressed, could be assigned to three principal metabolic pathways: Photosynthesis, Cellular energetic metabolism and Protein turnover. Furthermore, pathways related to Signaling and Stress response, though similar in their overall expression between the two depths, showed different protein compositions.

### Photosynthetic processes

Light availability, both intensity and quality, influences directly and indirectly chloroplast metabolism (Jiao et al., 2007). The modulation of photosynthetic machinery is critical in the short term (day by day) and long-term (season, years) adaptation to environmental light. In photosynthetic organisms, the adaptation to different light conditions happens through adjustments of cellular homeostasis to maintain a balance between energy supply (light harvesting and electron transport) and consumption (cellular metabolism). The regulation of these mechanisms involves changes in the expression levels of both mRNA and mature proteins. During the sampling, the irradiance at the deep stand was about 1/10 of the irradiance present at the shallow stand, with values that are very close to the theoretical minimum light requirement estimated for *P. oceanica* (~9–16% of surface irradiance, Lee et al., 2007). Hence, many genes and proteins belonging to the photosynthetic machinery resulted differentially regulated between stands, in order to perform photosynthesis under such different light conditions.

Transcriptional and proteomic profiles showed high differentiation on Chlorophyll a-b-binding (*Cab*) proteins between the two depths. An increase of Chlorophyll concentration under low-light was reported for other seagrasses (Dennison, 1990; Sharon et al., 2011). In *P. oceanica* chlorophyll rate was reported to vary not only along the depth gradient, but also during different seasons (Pirc, 1986). In addition, differences among *Cab* proteins identified between depths, suggest that in *P. oceanica* different *Cab* proteins are utilized for the assembly of the antenna complex, in response to specific photo-acclimation processes. It seems that, to prevent photo-damage due to high-light, plants evolved different strategies, such as the shrinking of PSII antenna size (Escoubas et al., 1995) and thermal dissipation (Elrad et al., 2002). Changes in antenna pigments compositions in low- light were also suggested for *P. oceanica* and for other seagrasses by Casazza and Mazzella (2002).

The relative quantity of transcripts and proteins recognized in this study also suggests an increase in PSII and PSI transcripts in deep plants in respect to the shallow ones (especially as regards as PSI). Photosynthetic-organisms balance electron flow between the two photosystems by modulating both antenna size and photosystem stoichiometry (Chitnis, 2001), in response to light intensity and quality. The redox status of the whole cell and of the chloroplast and the ratio between ATP and NADPH could also contribute in modulating PSI/II relative abundance (Chitnis, 2001). PSI/II ratio was found modified across depth also in the seagrass *Halophila stipulacea* (Sharon et al., 2011), in macroalgae (Fujita, 1997; Yamazaki et al., 2005) and cyanobacteria (Levitan et al., 2010) as to indicate that this could be a general photo-acclimatory mechanism. At the present, we are not able to explain the regulative mechanisms underlying this differential modulation between shallow and deep plants, but similar patterns of PSI/II ratio were already observed in shallow *P. oceanica* meadows growing under different light conditions (Mazzuca et al., 2009). Authors reported a reorganization of the thylakoid architecture under low-light conditions, that is consistent with the rearrangement between the two photosystems, since approximately 85% of PSII is located in the apprised domains of the grana and 64% of PSI is located in the stroma lamellae.

Another interesting hint suggested from our data for the *P. oceanica* photosynthetic acclimation involves the enzyme RuBisCo. The expression pattern of this enzyme between the two light conditions was different from the expectation: we measured, in fact, a similar content of this protein between shallow and deep stations, with a slightly higher abundance in low-light, especially for what concern the large subunit. This is in contrast with previous results, where Mazzuca et al. (2009) showed a clear decrease of the same protein in low-light condition in *P. oceanica*. The activity of RuBisCo responds to different environmental signals including light, changes in source-sin balance, temperature and circadian rhythms [reviewed in Portis (2003)]. However, regulation of RuBisCo is mediated, among others, by the activity of the chaperone Ribulose bisphosphate carboxylase/oxygenase activase A (RCA). This protein was identified in our collection as over-expressed, even if at low levels, in low-light condition. RCA is thought to have a key role in the regulation of photosynthesis under different environmental stress conditions (Portis, 2003) and during the daily cycle (Yin et al., 2010). In a recently study of Yamori et al. (2012) it was reported that in low-light condition, high expression of RCA contributes to maintain RuBisCo in high active state, helping in assuring high levels of CO_2_ assimilation also under shade conditions. These observations open the question regarding the real regulation mechanism of RuBisCo in *P. oceanica* in response to light, especially for what regards limiting light conditions.

### Cellular energetic metabolism

For what concerns respiration, an overall increase of related transcripts and proteins was recorded in shallow plants, probably related to the higher temperature present in respect to the deeper portion of the meadow plants [overview in Touchette and Burkholder (2000)]. Nevertheless, considering separately the regulation of each of the three main stages of the respiratory process, we see that glycolysis and electron transport chain steps were strongly enhanced in high light, while the tricarboxylic acid (TCA) cycle was higher in low light.

The understanding of the regulations of these pathways in plants is further complicated by the interactions between them and many other key elements (Fernie et al., 2004). Among the putative regulatory enzymes of mitochondrial activity (Bunney et al., 2001), a protein like 14-3-3 was recognized in our peptide collections. Collectively, plant 14-3-3s isoforms, which bind to phosphorylated client proteins to modulate their function, are implicated in an expanding catalogue of physiological functions and are affected by the extracellular and intracellular environment of the plant. They play a central role in the response to the plant extracellular environment, particularly environmental stress, pathogens, and light conditions (Denison et al., 2011).

### Stress response

Several transcripts encoding for proteins associated with stress response and plant defense were detected in low-light. Amongst these, metallothionein-like protein, which are implicated in metal tolerance in plants (Cobbett, 2000), Catalase and Oxygen-evolving enhancer proteins, which respond to reactive oxygen species (ROS) stress and are responsible for the breakdown of hydrogen peroxide to oxygen and water (Blokhina, 2003) and also the Cytochromes P450 family, which is implicated in detoxification. It is known that *P. oceanica* may accumulate metals from the sediment in its organs and tissues (Warnau et al., 1996; Schlacher-Hoenlinger and Schlacher, 1998) and the study by Giordani et al. (2000) have demonstrated that treatments with Mercury, Copper and Cadmium may induce the production of Metallothionein proteins in this species. Moreover, in the deep plants several transcripts encoding for Zinc finger domain stress-associated proteins and the 2-caffeic-acido-methyl transferase, were also found. The same proteins were also previously recognized (Mazzuca et al., 2009) in *P. oceanica* in similar environmental condition and associated to biotic and abiotic stress response (Cozza et al., 2006).

All these elements suggest that plants living in the deep stands are more sensitive to oxidative stress than plants growing in shallow stands, due to the higher investment by the former in maintaining basal metabolism and the consequent lower resources available for cell defense and repair. In addition, deep plants could also respond to exogenous oxidative stress due to the local distribution of stressing factors, which seem to be more important in the area of the bay where the deep stand is growing.

### Protein turnover

Many clones with sequence homology on components of the (Ub)Ubiquitin-26S proteasome pathway were identified in both ESTs collections. This degradation pathway is involved in the removal of abnormal polypeptides throughout normal protein turnover, and provides the degradation of enzymes and key regulatory factors of signal transduction, making it one of the most elaborate regulatory mechanisms in plants, allowing cells to respond rapidly to signal molecules and changes in environmental conditions (Gagne et al., 2004; Moon et al., 2004). Higher expression level of ubiquitin/26S proteins was already found in *P. oceanica* as consequence of plants acclimation to low-light conditions (Mazzuca et al., 2009). Three components of this complex witch appeared to be more expressed in high-light condition in comparison with low-light are involved in “protein-targeting”: the E3 ubiquitin-protein ligase, a U-box and RING-box protein and the SCF-E3, F-Box protein (Moon et al., 2004). The participation of SCFs in plant development is extensive, affecting processes such as hormone response, photo-morphogenesis, circadian rhythms, floral development, and senescence (Du et al., 2009). Moreover, several studies support that F-box proteins such as SCF E3, are also involved in phyA-mediated light signaling and in the regulation of circadian clock, making it possible that SCF proteins degrade a repressor of light response in preparation for light signals at dawn (Harmon and Kay, 2003).

Furthermore, plants growing at the different depths appear to respond not only to different environmental signals, but also to different endogenous signaling, such as hormones. In shallow plants, several component of the Ethylene signaling pathway were detected, such as the above mentioned F-box proteins. At present, information exists on the functions of a relatively small number of F-box proteins and most of these are involved in regulation of the hormone signaling pathway.

The role of the SCF is to degrade repressors of hormone response (auxin, GA, and JA), whereas in response to ethylene, the SCF degrades positive regulators in the absence of the hormone. The existing data strongly suggest that the principal control point of Ethylene signaling regulation is protein degradation via the ubiquitin/26S proteasome pathway (Potuschak et al., 2003; Kendrick and Chang, 2009). Ethylene is an important gaseous hormone's regulator in several plants processes, as (i) the regulation of endogenous rhythms, e.g., seed germination, plant growth, leaf expansion, root hair formation, fruit ripening, and timing of vegetative senescence and (ii) the transduction of environmental signaling, e.g., responses to abiotic stresses and pathogen attack (Potuschak et al., 2003; Raab et al., 2009). According to these indications, the different activity of ubiquitin-mediated proteolysis recognized between shallow and deep growing plants of *P. oceanica* could depend from the different seasonal timing at which they respond. Buia and Mazzella (1991) previously observed in the seagrasses *P. oceanica*, *Cymodocea nodosa* and *Zostera noltii*, a clear shift in life cycle between plants growing in shallow and deep stands in the Mediterranean Sea, with the effect that shallow plants (−5 m) complete their annual cycle in early summer, turning into senescence, while at the same time plants of the deep stands (−25 m) are fully growing. Our data about the photo-acclimation response of *P. oceanica* along the bathymetric gradient probably also reflect the different adjustments in life cycle during the year of plants growing at different depths. This allows plants to growth, optimizing the harvesting and the utilization of the available light in the different seasonal conditions and to minimize the negative effects due to photo-damage.

In conclusion, this study allowed to identify several regulatory networks and metabolic pathways involved in environmental signals response along the depth distribution of *P. oceanica*, and allowed to improve the available molecular resources, which is an important requisite for the application of eco-genomic approaches in this species.

### Conflict of interest statement

The authors declare that the research was conducted in the absence of any commercial or financial relationships that could be construed as a potential conflict of interest.

## Supplementary material

Figure S1 **RT-qPCR SSH library.** Expression levels of Pooc_B_c42 and Pooc_B_c 217 (y-axis, Mean ± SD) in *Posidonia oceanica* shoots collected at −25 m (deep-library). Shoots collected at −5 m (shallo-library) were used as control and the expression level of GOI in the control is represented in the figure by the x-axis. RT-qPCR data were normalized **(A)** with the best RGs in this experimental condition (EF1A, L23, NTUBC, Serra et al., 2012b), **(B)** using “universal” RGs (EF1A, NTUBC, 18S, and UBI) (^*^*p* < 0.05, ^***^*p* < 0.001). Additional information on primers sequences and RT-qPCR conditions in Serra et al. (2012b).

Table S1 **Additional statistics features of SSH–EST library**. Additional statistics features of SSH–ESTs *Posidonia oceanica* library.

Table S2a **List of Unigenes belonging to the shallow (high-light) library**. List of Unigenes belonging to the shallow (high-light) library. Unigenes name, their functional annotation with the *E*-value, number of ESTs identified (S_EST) and sequences lengths (Length) are indicated. For each sequence, the presence of putative ORF (open reading frame), SSRs (simple sequences repeats) and SNPs (single-nucleotide polymorphisms) are also showed.

Table S2b **List of Unigenes belonging to the deep (low-light) library**. List of Unigenes belonging to the deep (low-light) library. Unigenes name, their functional annotation with the *E*-value, number of ESTs identified (S_EST) and sequences lengths (Length) are indicated. For each sequence, the presence of putative ORF (open reading frame), SSRs (simple sequences repeats) and SNPs (single-nucleotide polymorphisms) are also showed.

Table S3a **List of peptides identified in the 1DE gel of proteins from shallow samples (S)**. List of peptides identified in the slices of 1DE gel of proteins from shallow samples (S), the protein attribution obtained with GPM and X!TANDEM sotfwares with the corresponding log(e) value, functional annotation obtained with TBLASTN search against Dr.Zompo database and corresponding *E*-value are shown.

Table S3b **List of peptides identified 1DE gel of proteins from deep samples (D)**. List of peptides identified in the slices of 1DE gel of proteins from deep samples (D), the protein attribution obtained with GPM and X!TANDEM softwares with the corresponding log(e) value, functional annotation obtained with TBLASTN search against Dr.Zompo database and corresponding *E*-value are shown.
