# Supplementary figures and images for: Acclimation to different depths by the marine angiosperm Posidonia oceanica: transcriptomic and proteomic profiles
Source: Front Plant Sci. 2013 Jun 17;4:195. doi: 10.3389/fpls.2013.00195 (PMC3683636; doi:10.3389/fpls.2013.00195)

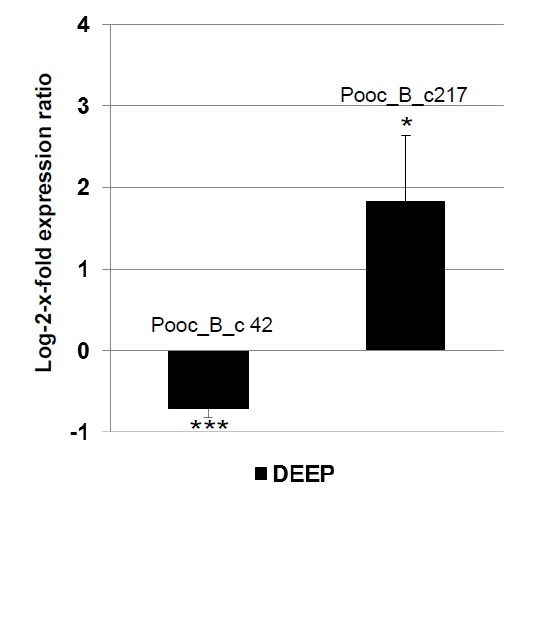

Supplement: Figure S1 — RT-qPCR SSH library. Expression levels of Pooc_B_c42 and Pooc_B_c 217 (y-axis, Mean ± SD) in Posidonia oceanica shoots collected at −25 m (deep-library). Shoots collected at −5 m (shallo-library) were used as control and the expression level of GOI in the control is represented in the figure by the x-axis. RT-qPCR data were normalized (A) with the best RGs in this experimental condition (EF1A, L23, NTUBC, Serra et al., 2012b), (B) using “universal” RGs (EF1A, NTUBC, 18S, and UBI) (*p < 0.05, ***p < 0.001). Additional information on primers sequences and RT-qPCR conditions in Serra et al. (2012b). [file Figure5.JPEG]
