# Supplementary material for: Acclimation to different depths by the marine angiosperm Posidonia oceanica: transcriptomic and proteomic profiles
Source: Front Plant Sci. 2013 Jun 17;4:195. doi: 10.3389/fpls.2013.00195 (PMC3683636; doi:10.3389/fpls.2013.00195)
Supplement: Table S1 — Additional statistics features of SSH–EST library. Additional statistics features of SSH–ESTs Posidonia oceanica library. [file DataSheet1.PDF]

Supplemental Table 1S. Additional statistics features of SSH -ESTs *Posidonia oceanica* library.

	N°
ESTs good sequences after trimming	2576
Contigs after EST assembly	486
Singletons after EST assembly	286
Unigenes (Contigs + Singletons)	772
Unigenes (Contigs + Singletons) with annotation	349
Unigenes (Contigs + Singletons) without annotation	423
Contigs with homology in Dr.Zompo	189
Without homology in Dr.Zompo	297
Unigenes with SNP	0
Unigenes with SSRs	36
