# Supplementary material for: Acclimation to different depths by the marine angiosperm Posidonia oceanica: transcriptomic and proteomic profiles
Source: Front Plant Sci. 2013 Jun 17;4:195. doi: 10.3389/fpls.2013.00195 (PMC3683636; doi:10.3389/fpls.2013.00195)
Supplement: Table S2a — List of Unigenes belonging to the shallow (high-light) library. List of Unigenes belonging to the shallow (high-light) library. Unigenes name, their functional annotation with the E-value, number of ESTs identified (S_EST) and sequences lengths (Length) are indicated. For each sequence, the presence of putative ORF (open reading frame), SSRs (simple sequences repeats) and SNPs (single-nucleotide polymorphisms) are also showed. [file DataSheet2.PDF]

Supplemental Table 2aS. List of Unigenes belonging to the Shallow (high-light) library. Unigenes name, their functional annotation with the E-value, Number of ESTs identified (S_EST) and sequences lengths (Length) are indicated. For each sequence, the presence of putative ORF (Open reading frame), SSRs (simple sequences repeats) and SNPs (single-nucleotide polymorphisms) are also showed.

Name	Functional Annotation	E-value	S_EST	Length	ORF	SSR	SNP
Pooc_B_c205	no hit		341	962 nt	x	0	0
Pooc_B_c132	Chlorophyll a-b binding protein 21, chloroplastic	0	78	723 nt	x	0	0
Pooc_B_c18	no hit		38	607 nt	x	0	0
Pooc_B_c444	F-box protein At5g67140	2.0 e-28	16	600 nt	x	0	0
Pooc_B_c209	no hit		15	508 nt	x	0	0
Pooc_B_c42	Cytochrome c oxidase subunit 1	3.0 e-14	15	866 nt	x	0	0
Pooc_B_c95	Heat shock cognate 70 kDa protein 2	2.0 e-11	12	484 nt	x	0	0
Pooc_B_c341	no hit		11	415 nt	x	0	0
Pooc_B_c449	lipid metabolic process		11	652 nt	x	0	0
Pooc_B_c130	no hit		10	580 nt	x	1	0
Pooc_B_c142	no hit		10	418 nt	x	0	0
Pooc_B_c250	Ketol-acid reductoisomerase, chloroplastic	0.0	10	622 nt	x	0	0
Pooc_B_c284	Photosystem II 10 kDa polypeptide, chloroplastic	2.0 e-35	10	458 nt	x	0	0
Pooc_B_c365	Cytochrome b6-f complex iron-sulfur subunit, chloroplastic	6.0 e-38	10	464 nt	x	0	0
Pooc_B_c198	no hit		9	569 nt	x	0	0
Pooc_B_c248	Cytochrome c oxidase polypeptide VIb	3.0 e-07	9	651 nt	x	0	0
Pooc_B_c36	ATP-dependent Clp protease proteolytic subunit-related protein 4, chloroplastic	1.96 e-39	9	434 nt	x	0	0
Pooc_B_c386	Chlorophyll a-b binding protein 151, chloroplastic	0	9	657 nt	x	0	0
Pooc_B_c434	ETO1-like protein 1	0	9	682 nt	x	0	0
Pooc_B_c208	no hit		8	365 nt	x	0	0
Pooc_B_c287	no hit		8	319 nt	x	0	0
Pooc_B_c362	no hit		8	508 nt	x	0	0
Pooc_B_c382	cytoplasmic membran e-bounded vesicle		8	443 nt	x	0	0
Pooc_B_c391	Probable ATP synthase 24 kDa subunit. mitochondrial	4.0 e-27	8	398 nt	x	0	0
Pooc_B_c116	nucleobase. nucleoside. nucleotide and nucleic acid metabolic process		7	567 nt	x	0	0
Pooc_B_c415	no hit		7	444 nt	x	0	0
Pooc_B_c44	U-box domain-containing protein 34	0	7	786 nt	x	0	0
Pooc_B_c110	no hit		6	600 nt	x	0	0
Pooc_B_c162	Regulator of ribonucleas e-like protein 3	0	6	498 nt	x	0	0
Pooc_B_c168	no hit		6	458 nt	x	0	0
Pooc_B_c215	no hit		6	673 nt	x	0	0
Pooc_B_c279	Putative syntaxin-24	1.0 e-06	6	457 nt	x	0	0
Pooc_B_c327	Ammonium transporter 1 member 2	2.0 e-16	6	674 nt	x	0	0
Pooc_B_c331	no hit		6	315 nt	x	0	0
Pooc_B_c370	Cytochrome P450 81D1	0	6	544 nt	x	0	0
Pooc_B_c417	no hit		6	311 nt	x	0	0

Pooc_B_c422	no hit		6	529 nt	x	0	0
Pooc_B_c428	Cystein e-rich receptor-like protein kinase 27	1.0 e-07	6	437 nt	x	0	0
Pooc_B_c486	Cytochrome b	6.0 e-38	6	616 nt	x	0	0
Pooc_B_c7	Acyl-CoA-binding protein	2.0 e-17	6	200 nt	x	0	0
Pooc_B_c97	Probable aquaporin PIP2-8	0	6	396 nt	x	0	0
Pooc_B_c118	Translocase of chloroplast 159. chloroplastic	5.0 e-27	5	499 nt	x	0	0
Pooc_B_c134	no hit		5	502 nt	x	0	0
Pooc_B_c146	biological_process		5	447 nt	x	0	0
Pooc_B_c229	Light regulated protein Lir1	2.2 e-10	5	511 nt	x	0	0
Pooc_B_c230	no hit		5	322 nt	x	0	0
Pooc_B_c314	no hit		5	419 nt	x	0	0
Pooc_B_c315	no hit		5	473 nt	x	0	0
Pooc_B_c337	Ethylen e-responsive transcription factor 5	8.0 e-19	5	257 nt	x	0	0
Pooc_B_c103	cytoplasmic chromosome		4	438 nt	x	0	0
Pooc_B_c112	Chlorophyll a-b binding protein CP24 10B. chloroplastic	5.0 e-15	4	500 nt	x	0	0
Pooc_B_c133	no hit		4	335 nt	x	0	0
Pooc_B_c136	no hit		4	313 nt	x	0	0
Pooc_B_c171	no hit		4	317 nt	x	0	0
Pooc_B_c174	no hit		4	867 nt	x	0	0
Pooc_B_c184	no hit		4	457 nt	x	0	0
Pooc_B_c211	no hit		4	316 nt	x	0	0
Pooc_B_c214	Positive match microRNA		4	530 nt	x	0	0
Pooc_B_c247	catalytic activity		4	259 nt	x	0	0
Pooc_B_c249	protein metabolic process		4	384 nt	x	0	0
Pooc_B_c255	Argininosuccinate synthase. chloroplastic	0.0	4	604 nt	x	0	0
Pooc_B_c301	no hit		4	392 nt	x	0	0
Pooc_B_c306	no hit		4	663 nt	x	0	0
Pooc_B_c310	Chlorophyll a-b binding protein 13. chloroplastic	1.0 e-34	4	288 nt	x	0	0
Pooc_B_c324	no hit		4	326 nt	x	0	0
Pooc_B_c325	Chaperone protein clpB 2	1.0 e-33	4	707 nt	x	0	0
Pooc_B_c414	no hit		4	276 nt	x	0	0
Pooc_B_c439	response to stress		4	462 nt	x	0	0
Pooc_B_c468	Fe(2+) transport protein 1	1.0 e-29	4	507 nt	x	0	0
Pooc_B_c473	Calmodulin	1.0 e-13	4	291 nt	x	1	0
Pooc_B_c481	no hit		4	318 nt	x	0	0
Pooc_B_c8	cytoplasmic vesicle		4	449 nt	x	0	0
Pooc_B_c91	no hit		4	426 nt	x	0	0
Pooc_B_c107	Thioredoxin M-type 4. chloroplastic	3.0 e-36	3	551 nt	x	0	0
Pooc_B_c126	F-box/LRR-repeat protein 5	8.0 e-38	3	513 nt	x	0	0
Pooc_B_c149	no hit		3	617 nt	x	0	0
Pooc_B_c163	catalytic activity		3	236 nt	x	0	0
Pooc_B_c172	no hit		3	456 nt	x	0	0
Pooc_B_c207	response to light stimulus		3	533 nt	x	0	0

Pooc_B_c228	Protein of unknown function (DUF3593)	5.6 e-30	3	521 nt	x	0	0
Pooc_B_c298	Chalcone synthase	1.0 e-34	3	481 nt	x	0	0
Pooc_B_c305	Cytochrome c oxidase subunit 2	3.0 e-21	3	443 nt	x	0	0
Pooc_B_c35	no hit		3	359 nt	x	0	0
Pooc_B_c366	no hit		3	412 nt	x	0	0
Pooc_B_c378	no hit		3	628 nt	x	0	0
Pooc_B_c384	no hit		3	164 nt		0	0
Pooc_B_c4	no hit		3	422 nt	x	0	0
Pooc_B_c426	Catalase isozyme 2	6.0 e-23	3	509 nt	x	0	0
Pooc_B_c43	no hit		3	400 nt	x	0	0
Pooc_B_c454	no hit		3	341 nt	x	0	0
Pooc_B_c485	no hit		3	522 nt	x	0	0
Pooc_B_c54	no hit		3	294 nt	x	0	0
Pooc_B_c58	Putative carboxyvinyl-carboxyphosphonate phosphorylmutase	0	3	718 nt	x	0	0
Pooc_B_c61	Acyl-CoA-binding protein	3.0 e-07	3	191 nt	x	0	0
Pooc_B_c1	10 kDa chaperonin	1.0 e-06	2	515 nt	x	0	0
Pooc_B_c10	Lipoxygenase. chloroplastic	3.0 e-38	2	455 nt	x	0	0
Pooc_B_c104	no hit		2	342 nt	x	0	0
Pooc_B_c109	no hit		2	308 nt	x	1	0
Pooc_B_c111	Cell division control protein 48 homolog D	1.0 e-10	2	301 nt	x	0	0
Pooc_B_c115	Two-component response regulator-like PRR37	5.0 e-06	2	505 nt	x	0	0
Pooc_B_c122	ATP-citrate synthase	2.0 e-12	2	318 nt	x	0	0
Pooc_B_c127	no hit		2	305 nt	x	0	0
Pooc_B_c137	no hit		2	309 nt	x	0	0
Pooc_B_c138	no hit		2	354 nt	x	0	0
Pooc_B_c14	no hit		2	679 nt	x	0	0
Pooc_B_c143	no hit		2	299 nt	x	1	0
Pooc_B_c15	no hit		2	367 nt	x	0	0
Pooc_B_c150	Pantoat e--beta-alanine ligase	7.0 e-10	2	296 nt	x	0	0
Pooc_B_c155	no hit		2	653 nt	x	0	0
Pooc_B_c158	cellular_component		2	294 nt	x	0	0
Pooc_B_c159	no hit		2	318 nt	x	0	0
Pooc_B_c165	chloroplast thylakoid		2	339 nt	x	0	0
Pooc_B_c166	no hit		2	350 nt	x	0	0
Pooc_B_c176	no hit		2	301 nt	x	1	0
Pooc_B_c179	no hit		2	309 nt	x	0	0
Pooc_B_c196	protein modification process		2	248 nt	x	0	0
Pooc_B_c20	no hit		2	232 nt		0	0
Pooc_B_c201	Fructos e-bisphosphate aldolase. chloroplastic	0	2	363 nt	x	0	0
Pooc_B_c203	no hit		2	250 nt	x	0	0
Pooc_B_c204	no hit		2	318 nt	x	0	0
Pooc_B_c213	no hit		2	226 nt	x	0	0
Pooc_B_c22	establishment of localization		2	289 nt	x	0	0

Pooc_B_c222	no hit		2	174 nt	x	0	0
Pooc_B_c227	TIP41-like protein	2.0 e-06	2	246 nt	x	0	0
Pooc_B_c232	Protein of unknown function (DUF789)	1.0 e-17	2	881 nt	x	1	0
Pooc_B_c235	50S ribosomal protein L35. chloroplastic	3.0 e-18	2	480 nt	x	0	0
Pooc_B_c24	Ethylene receptor	2.0 e-09	2	530 nt	x	0	0
Pooc_B_c25	no hit		2	535 nt	x	0	0
Pooc_B_c254	Nitrate reductase [NADH] 1	5.0 e-30	2	326 nt	x	0	0
Pooc_B_c256	no hit		2	333 nt	x	0	0
Pooc_B_c260	Uncharacterized TPR repeat-containing protein At1g05150	4.00 e-35	2	463 nt	x	0	0
Pooc_B_c267	RING-box protein 1a	0	2	640 nt	x	0	0
Pooc_B_c27	no hit		2	339 nt	x	0	0
Pooc_B_c270	Coatomer subunit alpha-1	0	2	650 nt	x	0	0
Pooc_B_c271	Photosystem I reaction center subunit III. chloroplastic	4.0 e-06	2	454 nt	x	0	0
Pooc_B_c273	cellular_component		2	325 nt	x	0	0
Pooc_B_c276	no hit		2	272 nt	x	0	0
Pooc_B_c29	Tetratricopeptide repeat	0.0001	2	259 nt	x	0	0
Pooc_B_c294	no hit		2	288 nt	x	0	0
Pooc_B_c295	no hit		2	252 nt	x	0	0
Pooc_B_c299	no hit		2	288 nt	x	0	0
Pooc_B_c300	molecular_function		2	379 nt	x	0	0
Pooc_B_c302	Pol polyprotein (Fragment)	2.0 e-06	2	502 nt	x	0	0
Pooc_B_c307	Zeaxanthin epoxidase. chloroplastic	8.0 e-07	2	515 nt	x	0	0
Pooc_B_c317	no hit		2	242 nt	x	1	0
Pooc_B_c326	DAG protein. chloroplastic	2.0 e-13	2	568 nt	x	0	0
Pooc_B_c333	no hit		2	354 nt	x	0	0
Pooc_B_c334	Glutaredoxin-C8	3.0 e-12	2	397 nt	x	0	0
Pooc_B_c339	PAR1 protein	0.0	2	829 nt	x	0	0
Pooc_B_c340	no hit		2	441 nt	x	0	0
Pooc_B_c345	Probable dolichyl pyrophosphate Glc1Man9GlcNAc2 alpha-1.3-glucosyltransferase	0	2	754 nt	x	0	0
Pooc_B_c347	Translationally-controlled tumor protein homolog	8.97 e-39	2	616 nt	x	1	0
Pooc_B_c350	Autophagy-related protein 8f	3.0 e-31	2	313 nt	x	0	0
Pooc_B_c357	no hit		2	368 nt	x	0	0
Pooc_B_c360	Chlorophyll a-b binding protein of LHCII type I. chloroplastic	0	2	487 nt	x	1	0
Pooc_B_c369	no hit		2	541 nt	x	0	0
Pooc_B_c37	no hit		2	252 nt	x	0	0
Pooc_B_c375	no hit		2	384 nt	x	0	0
Pooc_B_c389	Putative E3 ubiquitin-protein ligase ARI4	1.0 e-07	2	267 nt	x	0	0
Pooc_B_c394	no hit		2	370 nt	x	0	0
Pooc_B_c398	Apoptosis 1 inhibitor	3.0 e-06	2	625 nt	x	0	0
Pooc_B_c40	60S acidic ribosomal protein P2	3.0 e-19	2	677 nt	x	0	0
Pooc_B_c400	Nucleobas e-ascorbate transporter 6	2.00 e-36	2	408 nt	x	0	0
Pooc_B_c402	no hit		2	412 nt	x	0	0

Pooc_B_c404	no hit		2	242 nt	x	0	0
Pooc_B_c416	no hit		2	314 nt	x	0	0
Pooc_B_c418	metabolic process		2	546 nt	x	0	0
Pooc_B_c421	no hit		2	420 nt	x	0	0
Pooc_B_c427	no hit		2	454 nt	x	0	0
Pooc_B_c429	no hit		2	196 nt	x	0	0
Pooc_B_c437	no hit		2	332 nt	x	0	0
Pooc_B_c443	no hit		2	378 nt	x	0	0
Pooc_B_c446	no hit		2	403 nt	x	0	0
Pooc_B_c45	Flavonoid 3'-monooxygenase	6.0 e-17	2	257 nt	x	0	0
Pooc_B_c455	Transcription factor BIM2	3.0 e-13	2	526 nt	x	0	0
Pooc_B_c461	Cell division protease ftsH homolog 1. chloroplastic	0	2	400 nt	x	0	0
Pooc_B_c462	Chlorophyll a-b binding protein CP26. chloroplastic	4.06 e-39	2	490 nt	x	0	0
Pooc_B_c474	no hit		2	218 nt	x	0	0
Pooc_B_c475	no hit		2	200 nt	x	1	0
Pooc_B_c480	PP2A regulatory subunit TAP46	3.0 e-37	2	479 nt	x	0	0
Pooc_B_c482	SCO2-like protein RBE_0699	3.0 e-17	2	494 nt	x	0	0
Pooc_B_c483	Cytochrome b-c1 complex subunit Rieske	1.0 e-33	2	515 nt	x	0	0
Pooc_B_c484	metabolic process		2	439 nt	x	0	0
Pooc_B_c50	Olee1-like protein	7.0 e-06	2	428 nt	x	0	0
Pooc_B_c69	no hit		2	237 nt	x	0	0
Pooc_B_c71	no hit		2	459 nt	x	0	0
Pooc_B_c72	no hit		2	462 nt	x	0	0
Pooc_B_c73	no hit		2	492 nt	x	0	0
Pooc_B_c80	U3 small nucleolar ribonucleoprotein protein IMP3	2.0 e-18	2	409 nt	x	0	0
Pooc_B_c85	no hit		2	375 nt	x	0	0
Pooc_B_c87	60S ribosomal protein L3	7.0 e-13	2	244 nt	x	0	0
Pooc_B_c89	Transcription factor TRY	3.0 e-12	2	336 nt	x	0	0
Pooc_B_c9	Trans-cinnamate 4-monooxygenase	4.0 e-23	2	286 nt	x	0	0
Pooc_B_c93	Probable eukaryotic translation initiation factor 5-2	8.0 e-20	2	367 nt	x	0	0
Pooc_B_c99	no hit		2	118 nt		0	0
Pooc_B_c262	Caffeoyl-CoA O-methyltransferase	4.0 e-11	1	294 nt	x	0	0
Pooc_B_c293	Chlorophyll a-b binding protein 1. chloroplastic	3.0 e-14	1	580 nt	x	0	0
Pooc_B_c319	Metallothionein-like protein type 3	2.0 e-15	1	540 nt	x	0	0
Pooc_B_c447	Metallothionein-like protein	3.0 e-16	1	691 nt	x	1	0
Pooc_B_c5	no hit		1	258 nt	x	0	0
Pooc_B_rs1_A12_F	no hit		1	114 nt		0	0
Pooc_B_rs1_A8_R	Uncharacterized TPR repeat-containing protein At1g05150	1.0 e-10	1	384 nt	x	0	0
Pooc_B_rs1_B3_F	Heat shock cognate 70 kDa protein 2	6.0 e-11	1	255 nt	x	0	0
Pooc_B_rs1_C2_R	no hit		1	218 nt	x	0	0
Pooc_B_rs1_C3_R	no hit		1	334 nt	x	0	0
Pooc_B_rs1_E4_R	no hit		1	486 nt	x	0	0
Pooc_B_rs1_F7_F	Myristoyl-acyl carrier protein thioesterase. chloroplastic	3.0 e-21	1	319 nt	x	0	0

Pooc_B_rs1_F9_F			1	155 nt	x	0	0
Pooc_B_rs1_G10_R	no hit		1	438 nt	x	0	0
Pooc_B_rs1_G3_F	no hit		1	108 nt		0	0
Pooc_B_rs1_R7_R	no hit		1	204 nt		0	0
Pooc_B_rs10_A1_F	no hit		1	552 nt	x	0	0
Pooc_B_rs10_A10_F	no hit		1	105 nt		0	0
Pooc_B_rs10_A12_F	U-box domain-containing protein 34	0	1	592 nt	x	0	0
Pooc_B_rs10_B3_F	no hit		1	159 nt	x	0	0
Pooc_B_rs10_B5_F	no hit		1	388 nt	x	0	0
Pooc_B_rs10_C4_R	Iron-sulfur cluster assembly enzyme ISCU mitocondrial	1.0 e-27	1	499 nt	x	0	0
Pooc_B_rs10_C9_F	no hit		1	141 nt	x	0	0
Pooc_B_rs10_D7_F	no hit		1	176 nt		0	0
Pooc_B_rs10_E4_F	no hit		1	103 nt		0	0
Pooc_B_rs10_F4_F	no hit		1	370 nt	x	0	0
Pooc_B_rs10_F9_F	no hit		1	115 nt		0	0
Pooc_B_rs10_G10_R	no hit		1	267 nt	x	0	0
Pooc_B_rs10_G2_F/R	Indol e-3-acetic acid-induced protein ARG7	5.0 e-08	1	546 nt	x	0	0
Pooc_B_rs10_G4_F	no hit		1	261 nt	x	0	0
Pooc_B_rs10_H3_F	no hit		1	274 nt	x	0	0
Pooc_B_rs10_R12_R	no hit		1	135 nt		0	0
Pooc_B_rs2_A2_R	no hit		1	187 nt	x	0	0
Pooc_B_rs2_B4_R	no hit		1	280 nt	x	0	0
Pooc_B_rs2_C5_F	no hit		1	126 nt	x	0	0
Pooc_B_rs2_C9_R	Chlorophyll a-b binding protein 40. chloroplastic	1.0 e-32	1	463 nt	x	0	0
Pooc_B_rs2_E10_R	Chlorophyll a-b binding protein 151. chloroplastic	3.0 e-36	1	442 nt	x	0	0
Pooc_B_rs2_G5_F	no hit		1	291 nt	x	0	0
Pooc_B_rs2_G5_R	no hit		1	273 nt	x	0	0
Pooc_B_rs2_H3_F	no hit		1	205 nt	x	0	0
Pooc_B_rs2_H7_R	no hit		1	230 nt	x	0	0
Pooc_B_rs2_H9_F/R	no hit		1	164 nt	x	0	0
Pooc_B_rs3_B11_F	Regulator of ribonucleas e-like protein 2	5.0 e-21	1	269 nt	x	0	0
Pooc_B_rs3_B4_R	no hit		1	411 nt	x	0	0
Pooc_B_rs3_B5_R	no hit		1	335 nt	x	0	0
Pooc_B_rs3_C12_R	no hit		1	462 nt	x	0	0
Pooc_B_rs3_D4_R	no hit		1	103 nt		0	0
Pooc_B_rs3_D5_R	no hit		1	103 nt		0	0
Pooc_B_rs3_F6_F	no hit		1	417 nt	x	0	0
Pooc_B_rs3_G12_R	no hit		1	421 nt	x	0	0
Pooc_B_rs3_G3_R	no hit		1	257 nt	x	0	0
Pooc_B_rs3_H2_R	no hit		1	301 nt	x	0	0
Pooc_B_rs3_R6_R	no hit		1	380 nt	x	0	0
Pooc_B_rs3_R9_R	no hit		1	124 nt	x	0	0
Pooc_B_rs4_A11_F	no hit		1	272 nt	x	0	0

Pooc_B_rs4_B12_F	DEAD-box ATP-dependent RNA helicase	2.0 e-16	1	765 nt	x	0	0
Pooc_B_rs4_B12_R	no hit		1	389 nt	x	0	0
Pooc_B_rs4_C12_F	no hit		1	467 nt	x	0	0
Pooc_B_rs4_C8_F	no hit		1	113 nt		0	0
Pooc_B_rs4_D11_R	no hit		1	493 nt	x	0	0
Pooc_B_rs4_D4_R	Cytochrome b6-f complex iron-sulfur subunit. chloroplastic	5.0 e-37	1	483 nt	x	0	0
Pooc_B_rs4_D9_F/R	no hit		1	340 nt	x	0	0
Pooc_B_rs4_E12_R	Translocase of chloroplast 159. chloroplastic	5.0 e-13	1	359 nt	x	0	0
Pooc_B_rs4_G11_F	no hit		1	446 nt	x	0	0
Pooc_B_rs4_G8_R	Uncharacterized TPR repeat-containing protein At1g05150	1.0 e-12	1	399 nt	x	0	0
Pooc_B_rs4_H8_R	F-box protein At5g67140	2.0 e-18	1	399 nt	x	0	0
Pooc_B_rs5_A1_R	no hit		1	350 nt	x	0	0
Pooc_B_rs5_A11_F	Regulator of ribonucleas e-like protein 2	1.0 e-23	1	331 nt	x	0	0
Pooc_B_rs5_A9_F	no hit		1	188 nt	x	0	0
Pooc_B_rs5_B1_F/R	Thioredoxin-X. chloroplastic	9.0 e-28	1	512 nt	x	0	0
Pooc_B_rs5_C12_F /R	Chlorophyll a-b binding protein 151. chloroplastic	4.0 e-23	1	611 nt	x	0	0
Pooc_B_rs5_D9_F	Dihydroflavonol-4-reductase	5.0 e-13	1	337 nt	x	0	0
Pooc_B_rs5_E11_F	no hit		1	123 nt		0	0
Pooc_B_rs5_E7_F	no hit		1	107 nt		0	0
Pooc_B_rs5_H1_F	no hit		1	129 nt	x	0	0
Pooc_B_rs6_A10_F	no hit		1	250 nt	x	0	0
Pooc_B_rs6_A8_R	no hit		1	80 nt		0	0
Pooc_B_rs6_A9_R	no hit		1	165 nt	x	0	0
Pooc_B_rs6_B1_R	no hit		1	116 nt		0	0
Pooc_B_rs6_B2_R	no hit		1	134 nt		0	0
Pooc_B_rs6_B5_R	no hit		1	103 nt		0	0
Pooc_B_rs6_C11_R	no hit		1	100 nt		0	0
Pooc_B_rs6_C7_R	no hit		1	129 nt	x	0	0
Pooc_B_rs6_D11_F	no hit		1	140 nt	x	0	0
Pooc_B_rs6_D4_F	no hit		1	297 nt	x	0	0
Pooc_B_rs6_D6_R	no hit		1	109 nt		0	0
Pooc_B_rs6_D8_R	no hit		1	106 nt		0	0
Pooc_B_rs6_E7_F/R	DEAD-box ATP-dependent RNA helicase 45	9.00 e-35	1	469 nt	x	0	0
Pooc_B_rs6_F5_F	no hit		1	105 nt		0	0
Pooc_B_rs6_G10_F	no hit		1	111 nt		0	0
Pooc_B_rs6_G12_R	no hit		1	328 nt	x	0	0
Pooc_B_rs6_G6_R	no hit		1	124 nt		0	0
Pooc_B_rs6_G8_R	no hit		1	100 nt		0	0
Pooc_B_rs6_H2_F	no hit		1	86 nt		0	0
Pooc_B_rs6_H5_F	no hit		1	119 nt		0	0
Pooc_B_rs6_H6_F/R	no hit		1	144 nt	x	0	0
Pooc_B_rs6_R10_R	no hit		1	106 nt		0	0
Pooc_B_rs7_A2_R	no hit		1	146 nt	x	0	0

Pooc_B_rs7_A3_R	no hit		1	208 nt	x	0	0
Pooc_B_rs7_A4_R	no hit		1	259 nt	x	0	0
Pooc_B_rs7_A6_R	no hit		1	461 nt	x	0	0
Pooc_B_rs7_A9_R	no hit		1	482 nt	x	0	0
Pooc_B_rs7_B11_F	Chlorophyll a-b binding protein M9. chloroplastic	5.0 e-13	1	389 nt	x	0	0
Pooc_B_rs7_B4_F/R	Probable calcium-binding protein CML47	6.0 e-13	1	446 nt	x	0	0
Pooc_B_rs7_E1_F	Chlorophyll a-b binding protein M9. chloroplastic	2.0 e-12	1	325 nt	x	0	0
Pooc_B_rs7_E9_R	no hit		1	452 nt	x	0	0
Pooc_B_rs7_F12_F	Chlorophyll a-b binding protein 40. chloroplastic	8.0 e-25	1	460 nt	x	0	0
Pooc_B_rs7_F9_F	no hit		1	347 nt	x	0	0
Pooc_B_rs7_G3_R	no hit		1	108 nt		0	0
Pooc_B_rs7_G9_R	no hit		1	348 nt	x	0	0
Pooc_B_rs7_H3_R	no hit		1	116 nt		0	0
Pooc_B_rs7_R12_R	Chlorophyll a-b binding protein M9. chloroplastic	1.0 e-23	1	439 nt	x	0	0
Pooc_B_rs7_R4_R	no hit		1	123 nt	x	0	0
Pooc_B_rs8_A4_F	no hit		1	157 nt	x	0	0
Pooc_B_rs8_A8_R	no hit		1	362 nt	x	0	0
Pooc_B_rs8_C3_R	no hit		1	325 nt	x	0	0
Pooc_B_rs8_D12_R	no hit		1	421 nt	x	0	0
Pooc_B_rs8_D2_R	ETO1-like protein 1	0	1	586 nt	x	0	0
Pooc_B_rs8_E12_R	no hit		1	533 nt	x	0	0
Pooc_B_rs8_G2_R	no hit		1	465 nt	x	0	0
Pooc_B_rs8_H11_FR	no hit		1	499 nt	x	0	0
Pooc_B_rs8_H6_R	no hit		1	308 nt	x	0	0
Pooc_B_rs8_H7_F/R	no hit		1	387 nt	x	0	0
Pooc_B_rs8_H9_R	no hit		1	409 nt	x	0	0
Pooc_B_rs8_R2_R	Chlorophyll a-b binding protein. chloroplastic	3.0 e-14	1	371 nt	x	0	0
Pooc_B_rs9_A5_R	no hit		1	499 nt	x	0	0
Pooc_B_rs9_A7_R	no hit		1	517 nt	x	0	0
Pooc_B_rs9_C9_R	no hit		1	134 nt	x	0	0
Pooc_B_rs9_D12_R	no hit		1	510 nt	x	1	0
Pooc_B_rs9_D7_R	no hit		1	473 nt	x	0	0
Pooc_B_rs9_E10_R	no hit		1	166 nt	x	0	0
Pooc_B_rs9_E4_F	no hit		1	451 nt	x	0	0
Pooc_B_rs9_E9_F	no hit		1	165 nt	x	0	0
Pooc_B_rs9_E9_R	no hit		1	114 nt		0	0
Pooc_B_rs9_F12_F	no hit		1	443 nt	x	0	0
Pooc_B_rs9_G3_F	no hit		1	450 nt	x	0	0
Pooc_B_rs9_G3_R	no hit		1	458 nt	x	0	0
Pooc_B_rs9_G6_R	Cytochrome c oxidase polypeptide VIb	9.0 e-08	1	314 nt	x	0	0
Pooc_B_rs9_H10_R	no hit		1	494 nt	x	0	0
Pooc_B_rs9_H11_FR	no hit		1	437 nt	x	0	0
Pooc_B_rs9_H12_R	no hit		1	105 nt		0	0

Pooc_B_rs9_H3_F	N(2).N(2)-dimethylguanosine tRNA methyltransferase	1.0 e-12	1	574 nt	x	0	0
Pooc_B_rs9_H8_R	no hit		1	511 nt	x	0	0
Pooc_B_rs9_R9_R	no hit		1	102 nt		0	0
