# Supplementary material for: Acclimation to different depths by the marine angiosperm Posidonia oceanica: transcriptomic and proteomic profiles
Source: Front Plant Sci. 2013 Jun 17;4:195. doi: 10.3389/fpls.2013.00195 (PMC3683636; doi:10.3389/fpls.2013.00195)
Supplement: Table S2b — List of Unigenes belonging to the deep (low-light) library. List of Unigenes belonging to the deep (low-light) library. Unigenes name, their functional annotation with the E-value, number of ESTs identified (S_EST) and sequences lengths (Length) are indicated. For each sequence, the presence of putative ORF (open reading frame), SSRs (simple sequences repeats) and SNPs (single-nucleotide polymorphisms) are also showed. [file DataSheet3.PDF]

Supplemental Table 2bS. List of Unigenes belonging to the Deep (low-light) library. Unigenes name. their functional annotation with the E-value. Number of ESTs identified (D_EST) and sequences lengths (Length) are indicated. For each sequence. the presence of putative ORF (Open reading frame). SSRs (simple sequences repeats) and SNPs (single-nucleotide polymorphisms) are also showed.

Name	Functional Annotation	E-value	D_EST	Length	ORF	SSRs	SNPs
Pooc_B_c132	Chlorophyll a-b binding protein 21. chloroplastic	0	75	723 nt	x	0	0
Pooc_B_c205	no hit		19	962 nt	x	0	0
Pooc_B_c386	Chlorophyll a-b binding protein 151. chloroplastic	0	17	657 nt	x	0	0
Pooc_B_c217	cellular_component		17	646 nt	x	0	0
Pooc_B_c142	no hit		12	418 nt	x	0	0
Pooc_B_c97	Probable aquaporin PIP2-8	0	12	396 nt	x	0	0
Pooc_B_c182	Protochlorophyllide reductase. chloroplastic	1.0 e-20	12	398 nt	x	0	0
Pooc_B_c246	no hit		11	320 nt	x	0	0
Pooc_B_c259	no hit		11	331 nt	x	0	0
Pooc_B_c262	Caffeoyl-CoA O-methyltransferase	4.0 e-11	10	294 nt	x	0	0
Pooc_B_c304	Protein of unknown function (DUF707)	7.6 e-36	10	828 nt	x	0	0
Pooc_B_c376	Universal stress protein A-like protein	3.0 e-07	10	620 nt	x	0	0
Pooc_B_c231	biological_process		9	359 nt	x	0	0
Pooc_B_c275	cellular_component		9	773 nt	x	0	0
Pooc_B_c421	no hit		8	420 nt	x	0	0
Pooc_B_c447	Metallothionein-like protein	3.0 e-16	8	691 nt	x	1	0
Pooc_B_c286	Protein of unknown function (DUF579)	6.5 e-21	8	272 nt	x	0	0
Pooc_B_c438	29 kDa ribonucleoprotein A. chloroplastic	2.0 e-34	8	788 nt	x	0	0
Pooc_B_c469	Signal peptide peptidase-like 2B	3.0 e-14	8	690 nt	x	1	0
Pooc_B_c70	no hit		8	338 nt	x	0	0
Pooc_B_c271	Photosystem I reaction center subunit III. chloroplastic	4.0 e-06	7	454 nt	x	0	0
Pooc_B_c274	plastid		7	739 nt	x	0	0
Pooc_B_c442	Serine--glyoxylate aminotransferase	0	7	638 nt	x	1	0
Pooc_B_c68	no hit		7	401 nt	x	1	0
Pooc_B_c84	Probable 1-deoxy-D-xylulose-5-phosphate synthase. chloroplastic	0	7	751 nt	x	0	0
Pooc_B_c108	no hit		6	496 nt	x	0	0
Pooc_B_c226	NADH dehydrogenase [ubiquinone] iron-sulfur protein 8. mitochondrial	3.0 e-26	6	463 nt	x	0	0
Pooc_B_c282	no hit		6	723 nt	x	0	0
Pooc_B_c31	Ferredoxin. chloroplastic	2.0 e-15	6	488 nt	x	0	0
Pooc_B_c312	Ferredoxin-thioredoxin reductase catalytic chain. chloroplastic	0	6	588 nt	x	0	0
Pooc_B_c349	no hit		6	574 nt	x	0	0
Pooc_B_c363	Mitochondrial import receptor subunit TOM40 homolog 1	0	6	637 nt	x	0	0
Pooc_B_c385	no hit		6	543 nt	x	0	0
Pooc_B_c409	Probable 6-phosphogluconolactonase 3. chloroplastic	7.0 e-21	6	523 nt	x	0	0
Pooc_B_c310	Chlorophyll a-b binding protein 13. chloroplastic	1.0 e-34	5	288 nt	x	0	0
Pooc_B_c144	no hit		5	555 nt	x	0	0
Pooc_B_c169	no hit		5	299 nt	x	0	0

Pooc_B_c17	no hit		5	375 nt	x	0	0	
Pooc_B_c194	BI1-like protein	1.0 e-11	5	639 nt	x	0	0	
Pooc_B_c216	Desiccation protectant protein Lea14 homolog	3.0 e-30	5	624 nt	x	0	0	
Pooc_B_c219	Magnesium-protoporphyrin IX monomethyl ester [oxidative] cyclase. chloroplastic	1.96 e-39	5	566 nt	x	0	0	
Pooc_B_c290	no hit		5	356 nt	x	0	0	
Pooc_B_c332	Stress up-regulated Nod 19	8.3 e-35	5	818 nt	x	0	0	
Pooc_B_c423	no hit		5	303 nt	x	0	0	
Pooc_B_c441	no hit		5	522 nt	x	0	0	
Pooc_B_c452	no hit		5	365 nt	x	0	0	
Pooc_B_c81	Oxygen-evolving enhancer protein 3-2. chloroplastic	3.0 e-37	5	613 nt	x	1	0	
Pooc_B_c229	Light regulated protein Lir1	2.2 e-10	4	511 nt	x	0	0	
Pooc_B_c122	ATP-citrate synthase	2.0 e-12	4	318 nt	x	0	0	
Pooc_B_c360	Chlorophyll a-b binding protein of LHCII type I. chloroplastic	0	4	487 nt	x	1	0	
Pooc_B_c319	Metallothionein-like protein type 3	2.0 e-15	4	540 nt	x	0	0	
Pooc_B_c101	response to endogenous stimulus		4	200 nt	x	0	0	
Pooc_B_c117	cellular_component		4	433 nt	x	0	0	
Pooc_B_c120	no hit		4	365 nt	x	0	0	
Pooc_B_c151	chloroplast thylakoid		4	370 nt	x	0	0	
Pooc_B_c164	no hit		4	445 nt	x	0	0	
Pooc_B_c173	no hit		4	310 nt	x	0	0	
Pooc_B_c206	Fructose-1.6-bisphosphatase. chloroplastic	8.0 e-30	4	343 nt	x	0	0	
Pooc_B_c220	Proteasome subunit alpha type-3	8.00 e-38	4	347 nt	x	0	0	
Pooc_B_c221	no hit		4	291 nt	x	0	0	
Pooc_B_c223	Tetraspanin family		4	644 nt	x	0	0	
Pooc_B_c225	Isocitrate dehydrogenase [NADP]. chloroplastic (Fragment)	2.0 e-24	4	582 nt	x	0	0	
Pooc_B_c236	cellular_component		4	419 nt	x	0	0	
Pooc_B_c237	no hit		4	285 nt	x	0	0	
Pooc_B_c239	no hit		4	307 nt	x	0	0	
Pooc_B_c263	no hit		4	252 nt	x	0	0	
Pooc_B_c266	no hit		4	422 nt	x	0	0	
Pooc_B_c268	Chlorophyll a-b binding protein 7. chloroplastic	4.0 e-10	4	266 nt	x	0	0	
Pooc_B_c278	no hit		4	527 nt	x	0	0	
Pooc_B_c28	no hit		4	366 nt	x	0	0	
Pooc_B_c280	no hit		4	319 nt	x	0	0	
Pooc_B_c3	no hit		4	341 nt	x	0	0	
Pooc_B_c30	Cytochrome P450 86A1	2.0 e-18	4	480 nt	x	0	0	
Pooc_B_c320	Chlorophyll a-b binding protein CP24 10A. chloroplastic	0	4	601 nt	x	0	0	
Pooc_B_c321	Transferase family	2.1 e-09	4	351 nt	x	0	0	
Pooc_B_c33	metabolic process		4	198 nt	x	0	0	
Pooc_B_c343	Magnesium-protoporphyrin IX monomethyl ester [oxidative] cyclase. chloroplastic	0	4	575 nt	x	0	0	

Pooc_B_c348	Flower-specific gamma-thionin	1.0 e-06	4	388 nt	x	0	0
Pooc_B_c351	no hit		4	431 nt	x	0	0
Pooc_B_c356	response to stress		4	274 nt	x	0	0
Pooc_B_c359	no hit		4	284 nt	x	0	0
Pooc_B_c367	no hit		4	268 nt	x	0	0
Pooc_B_c374	Calmodulin	0	4	860 nt	x	0	0
Pooc_B_c388	no hit		4	300 nt	x	0	0
Pooc_B_c393	acetyl-CoA metabolic process		4	490 nt	x	0	0
Pooc_B_c395	no hit		4	241 nt	x	0	0
Pooc_B_c401	no hit		4	623 nt	x	0	0
Pooc_B_c403	lipid metabolic process		4	483 nt	x	0	0
Pooc_B_c41	Chalcone synthase 2	1.0 e-22	4	415 nt	x	0	0
Pooc_B_c420	Glutamine synthetase cytosolic isozyme	4.0 e-24	4	575 nt	x	0	0
Pooc_B_c430	no hit		4	354 nt	x	0	0
Pooc_B_c432	no hit		4	174 nt	x	0	0
Pooc_B_c440	14 kDa proline-rich protein DC2.15	3.0 e-09	4	239 nt	x	0	0
Pooc_B_c457	Mitochondrial folate transporter/carrier	6.0 e-11	4	356 nt	x	0	0
Pooc_B_c458	cytoplasmic membrane-bounded vesicle		4	480 nt	x	0	0
Pooc_B_c459	no hit		4	123 nt	x	0	0
Pooc_B_c465	no hit		4	316 nt	x	0	0
Pooc_B_c471	Protein of unknown function (DUF1313)	2.4 e-14	4	189 nt	x	0	0
Pooc_B_c472	phosphoprotein phosphatase activity	4.0 e-09	4	715 nt	x	0	0
Pooc_B_c478	no hit		4	245 nt	x	0	0
Pooc_B_c83	Oxygen-evolving enhancer protein 2. chloroplastic	0	4	532 nt	x	0	0
Pooc_B_c98	Naringenin.2-oxoglutarate 3-dioxygenase (Fragment)	1.0 e-22	4	216 nt	x	0	0
Pooc_B_c213	no hit		3	226 nt	x	0	0
Pooc_B_c12	Probable beta-1.3-galactosyltransferase 17	9.0 e-10	3	363 nt	x	0	0
Pooc_B_c13	no hit		3	414 nt	x	1	0
Pooc_B_c180	Protochlorophyllide reductase. chloroplastic	0	3	609 nt	x	0	0
Pooc_B_c189	Uncharacterized protein ART3	3.0 e-07	3	303 nt	x	0	0
Pooc_B_c190	proteolysis		3	337 nt	x	0	0
Pooc_B_c191	Dual specificity protein phosphatase 12	3.0 e-14	3	634 nt	x	0	0
Pooc_B_c192	no hit		3	452 nt	x	0	0
Pooc_B_c2	no hit		3	287 nt	x	0	0
Pooc_B_c218	transferase activity		3	312 nt	x	0	0
Pooc_B_c23	S-acyltransferase TIP1	0	3	906 nt	x	0	0
Pooc_B_c264	no hit		3	109 nt		0	0
Pooc_B_c292	Clavaminate synthase-like protein At3g21360	3.0 e-07	3	472 nt	x	0	0
Pooc_B_c313	40S ribosomal protein S15	0	3	622 nt	x	0	0
Pooc_B_c358	Coatomer subunit beta'-1	0	3	831 nt	x	0	0
Pooc_B_c361	no hit		3	460 nt	x	0	0
Pooc_B_c373	no hit		3	319 nt	x	0	0
Pooc_B_c380	no hit		3	385 nt	x	0	0

Pooc_B_c436	no hit		3	239 nt	x	0	0
Pooc_B_c477	no hit		3	298 nt	x	0	0
Pooc_B_c51	Alanyl-tRNA synthetase. mitochondrial	0	3	696 nt	x	0	0
Pooc_B_c76	Hydrophobic protein RCI2B	4.0 e-15	3	235 nt	x	0	0
Pooc_B_c95	Heat shock cognate 70 kDa protein 2	2.0 e-11	2	484 nt	x	0	0
Pooc_B_c365	Cytochrome b6-f complex iron-sulfur subunit. chloroplastic	6.0 e-38	2	464 nt	x	0	0
Pooc_B_c126	F-box/LRR-repeat protein 5	8.0 e-38	2	513 nt	x	0	0
Pooc_B_c143	no hit		2	299 nt	x	1	0
Pooc_B_c339	PAR1 protein	0.0	2	829 nt	x	0	0
Pooc_B_c462	Chlorophyll a-b binding protein CP26. chloroplastic	4.06 e-39	2	490 nt	x	0	0
Pooc_B_c5	no hit		2	258 nt	x	0	0
Pooc_B_c100	Glyoxysomal fatty acid beta-oxidation multifunctional protein MFP-a	0	2	438 nt	x	0	0
Pooc_B_c102	no hit		2	374 nt	x	0	0
Pooc_B_c105	26S protease regulatory subunit S10B	0	2	728 nt	x	0	0
Pooc_B_c106	no hit		2	481 nt	x	0	0
Pooc_B_c11	no hit		2	179 nt	x	0	0
Pooc_B_c113	no hit		2	342 nt	x	0	0
Pooc_B_c114	catalytic activity		2	699 nt	x	0	0
Pooc_B_c119	no hit		2	274 nt	x	0	0
Pooc_B_c121	no hit		2	202 nt	x	0	0
Pooc_B_c123	Protein of unknown function (DUF581)	1.3 e-09	2	578 nt	x	1	0
Pooc_B_c124	Chlorophyll a-b binding protein P4. chloroplastic	2.0 e-30	2	460 nt	x	0	0
Pooc_B_c125	protein kinase activity		2	568 nt	x	0	0
Pooc_B_c128	no hit		2	327 nt	x	1	0
Pooc_B_c129	no hit		2	490 nt	x	0	0
Pooc_B_c131	no hit		2	264 nt	x	0	0
Pooc_B_c135	2-Cys peroxiredoxin BAS1-like. chloroplastic	0	2	458 nt	x	0	0
Pooc_B_c139	Peroxidase 42	6.0 e-17	2	325 nt	x	0	0
Pooc_B_c140	Alpha-1.4-glucan-protein synthase [UDP-forming]	0	2	556 nt	x	0	0
Pooc_B_c141	Bifunctional monodehydroascorbate reductase and carbonic anhydrase nectarin-3	1.0 e-07	2	156 nt	x	0	0
Pooc_B_c145	no hit		2	253 nt	x	0	0
Pooc_B_c147	Ferredoxin. chloroplastic	1.0 e-15	2	312 nt	x	0	0
Pooc_B_c148	no hit		2	336 nt	x	1	0
Pooc_B_c152	Peroxiredoxin Q. chloroplastic	0	2	599 nt	x	0	0
Pooc_B_c153	no hit		2	208 nt	x	0	0
Pooc_B_c154	no hit		2	412 nt	x	0	0
Pooc_B_c156	no hit		2	150 nt	x	0	0
Pooc_B_c157	no hit		2	283 nt	x	0	0
Pooc_B_c16	no hit		2	245 nt	x	0	0
Pooc_B_c160	Probable phospholipid hydroperoxide glutathione peroxidase	8.0 e-19	2	307 nt	x	0	0
Pooc_B_c161	Putative selenium-binding protein	0	2	514 nt	x	0	0
Pooc_B_c167	lipid metabolic process		2	302 nt	x	0	0

Pooc_B_c170	Probable receptor-like protein kinase At3g46290	5.0 e-40	2	402 nt	x	0	0
Pooc_B_c175	Probable flavin-containing monooxygenase 1	7.0 e-15	2	350 nt	x	0	0
Pooc_B_c177	Phytosulfokines	9.0 e-12	2	546 nt	x	0	0
Pooc_B_c178	UPF0041 protein C24B11.09	2.0 e-10	2	272 nt	x	0	0
Pooc_B_c181	Cyclin-K	5.0 e-08	2	264 nt	x	0	0
Pooc_B_c183	S-adenosylmethionine synthetase	1.0 e-22	2	241 nt	x	0	0
Pooc_B_c185	no hit		2	223 nt	x	0	0
Pooc_B_c186	no hit		2	209 nt	x	0	0
Pooc_B_c187	no hit		2	269 nt	x	0	0
Pooc_B_c188	Potassium channel AKT6	2.0 e-07	2	245 nt	x	0	0
Pooc_B_c19	no hit		2	258 nt	x	0	0
Pooc_B_c193	5'-adenylylsulfate reductase 3. chloroplastic	0	2	549 nt	x	0	0
Pooc_B_c195	Oxygen-evolving enhancer protein 1. chloroplastic	9.95 e-39	2	314 nt	x	0	0
Pooc_B_c197	oxidoreductase activity		2	438 nt	x	0	0
Pooc_B_c199	26S proteasome non-ATPase regulatory subunit 14	2.0 e-33	2	375 nt	x	0	0
Pooc_B_c200	no hit		2	354 nt	x	0	0
Pooc_B_c202	no hit		2	342 nt	x	0	0
Pooc_B_c21	no hit		2	444 nt	x	0	0
Pooc_B_c210	membrane-bounded organelle		2	646 nt	x	0	0
Pooc_B_c212	Photosystem II 22 kDa protein. chloroplastic	5.0 e-11	2	365 nt	x	0	0
Pooc_B_c224	no hit		2	330 nt	x	1	0
Pooc_B_c233	Probable inorganic phosphate transporter 1-8	1.00 e-37	2	385 nt	x	0	0
Pooc_B_c234	Elongation factor 1-gamma 1	2.0 e-09	2	316 nt	x	0	0
Pooc_B_c238	no hit		2	517 nt	x	0	0
Pooc_B_c240	Photosystem II reaction centre W protein (PsbW)	1.3 e-33	2	348 nt	x	0	0
Pooc_B_c241	Glutaredoxin	4.0 e-39	2	604 nt	x	0	0
Pooc_B_c242	no hit		2	349 nt	x	0	0
Pooc_B_c243	one-carbon metabolic process		2	617 nt	x	0	0
Pooc_B_c244	B12D protein	1.2 e-08	2	284 nt	x	0	0
Pooc_B_c245	Transformation/transcription domain-associated protein	4.0 e-07	2	470 nt	x	0	0
Pooc_B_c251	Putative lactoylglutathione lyase	0.0	2	648 nt	x	0	0
Pooc_B_c252	no hit		2	244 nt	x	0	0
Pooc_B_c253	Protein of unknown function (DUF581)	1.5 e-08	2	410 nt	x	0	0
Pooc_B_c257	no hit		2	371 nt	x	0	0
Pooc_B_c258	no hit		2	281 nt	x	0	0
Pooc_B_c26	no hit		2	165 nt	x	0	0
Pooc_B_c261	Zinc finger protein 1	6.0 e-06	2	425 nt	x	0	0
Pooc_B_c265	no hit		2	322 nt	x	0	0
Pooc_B_c269	no hit		2	374 nt	x	0	0
Pooc_B_c272	Chlorophyll a-b binding protein CP29.1. chloroplastic	2.0 e-34	2	318 nt	x	0	0
Pooc_B_c277	Glutamate decarboxylase 2	6.0 e-30	2	418 nt	x	0	0

Pooc_B_c281	Tubulin alpha chain	9.00 e-37	2	289 nt	x	0	0
Pooc_B_c283	no hit		2	538 nt	x	1	0
Pooc_B_c285	no hit		2	277 nt	x	0	0
Pooc_B_c288	protein kinase activity		2	654 nt	x	0	0
Pooc_B_c289	no hit		2	310 nt	x	0	0
Pooc_B_c291	Peroxisome assembly factor 2	4.0 e-07	2	423 nt	x	0	0
Pooc_B_c296	Oxygen-evolving enhancer protein 1. chloroplastic	2.0 e-24	2	276 nt	x	0	0
Pooc_B_c297	no hit		2	328 nt	x	0	0
Pooc_B_c303	Glycine rich protein family	1.3 e-08	2	385 nt	x	0	0
Pooc_B_c308	Serine/threonine-protein kinase HT1	3.0 e-31	2	278 nt	x	0	0
Pooc_B_c309	no hit		2	165 nt	x	0	0
Pooc_B_c311	cellular_component		2	389 nt	x	0	0
Pooc_B_c316	Phospholipase D alpha 1	3.0 e-09	2	554 nt	x	0	0
Pooc_B_c318	Protein transport protein Sec61 subunit alpha	0	2	458 nt	x	0	0
Pooc_B_c32	no hit		2	262 nt	x	0	0
Pooc_B_c322	Histone H3.3	3.0 e-31	2	383 nt	x	0	0
Pooc_B_c323	Germin-like protein subfamily 2 member 4	0	2	465 nt	x	0	0
Pooc_B_c328	no hit		2	380 nt	x	0	0
Pooc_B_c329	Positive match microRNA		2	267 nt	x	0	0
Pooc_B_c330	Putative metal tolerance protein C3	0	2	719 nt	x	0	0
Pooc_B_c335	no hit		2	282 nt	x	0	0
Pooc_B_c336	no hit		2	250 nt	x	0	0
Pooc_B_c338	no hit		2	330 nt	x	0	0
Pooc_B_c34	molecular_function		2	320 nt	x	0	0
Pooc_B_c342	no hit		2	453 nt	x	0	0
Pooc_B_c344	Heat shock cognate 70 kDa protein 2	2.0 e-39	2	373 nt	x	0	0
Pooc_B_c346	Harpin-induced protein 1 (Hin1)		2	278 nt	x	0	0
Pooc_B_c352	Fasciclin-like arabinogalactan protein 16	1.0 e-23	2	396 nt	x	0	0
Pooc_B_c353	no hit		2	312 nt	x	0	0
Pooc_B_c354	regulation of nucleotide metabolic process		2	240 nt	x	0	0
Pooc_B_c355	BEL1-like homeodomain protein 1	7.0 e-12	2	725 nt	x	0	0
Pooc_B_c364	Chlorophyll a-b binding protein 6A. chloroplastic	2.0 e-08	2	259 nt	x	0	0
Pooc_B_c368	60S ribosomal protein L10	0	2	490 nt	x	1	0
Pooc_B_c371	no hit		2	625 nt	x	0	0
Pooc_B_c372	Ferrochelatase-2. chloroplastic	6.0 e-38	2	313 nt	x	0	0
Pooc_B_c377	no hit		2	229 nt		0	0
Pooc_B_c379	cellular_component		2	666 nt	x	0	0
Pooc_B_c38	Putative uncharacterized protein YCR013C	5.0 e-10	2	563 nt	x	0	0
Pooc_B_c381	no hit		2	345 nt	x	0	0
Pooc_B_c383	no hit		2	260 nt	x	0	0
Pooc_B_c387	no hit		2	155 nt	x	0	0
Pooc_B_c39	Peptidyl-tRNA hydrolase. chloroplastic	7.0 e-24	2	219 nt	x	0	0

Pooc_B_c390	Actin	1.0 e-16	2	310 nt	x	0	0
Pooc_B_c392	no hit		2	343 nt	x	0	0
Pooc_B_c396	no hit		2	243 nt	x	0	0
Pooc_B_c397	Dihydroxy-acid dehydratase	5.0 e-09	2	368 nt	x	0	0
Pooc_B_c399	no hit		2	470 nt	x	0	0
Pooc_B_c405	no hit		2	316 nt	x	0	0
Pooc_B_c406	no hit		2	765 nt	x	1	0
Pooc_B_c407	Probable xyloglucan endotransglucosylase/hydrolase protein 27	2.0 e-25	2	541 nt	x	0	0
Pooc_B_c408	Chlorophyll a-b binding protein 1B. chloroplastic	2.00 e-37	2	439 nt	x	0	0
Pooc_B_c410	60S ribosomal protein L29-1	3.0 e-17	2	315 nt	x	0	0
Pooc_B_c411	transport		2	593 nt	x	0	0
Pooc_B_c412	Basic endochitinase CHB4	6.00 e-36	2	512 nt	x	0	0
Pooc_B_c413	ADP-ribosylation factor	3.0 e-18	2	332 nt	x	0	0
Pooc_B_c419	GTP-binding nuclear protein Ran/TC4	0	2	370 nt	x	0	0
Pooc_B_c424	cellular_component		2	473 nt	x	0	0
Pooc_B_c425	50S ribosomal protein L29. chloroplastic	1.0 e-23	2	354 nt	x	0	0
Pooc_B_c431	no hit		2	328 nt	x	1	0
Pooc_B_c433	transport		2	465 nt	x	0	0
Pooc_B_c435	no hit		2	532 nt	x	0	0
Pooc_B_c445	Chlorophyll a-b binding protein CP29.2. chloroplastic	5.0 e-22	2	336 nt	x	0	0
Pooc_B_c448	Salt tolerance protein	9.0 e-18	2	661 nt	x	0	0
Pooc_B_c450	Actin-related protein 5	3.0 e-14	2	305 nt	x	0	0
Pooc_B_c451	Probable proteasome inhibitor	9.0 e-07	2	407 nt	x	0	0
Pooc_B_c453	Eukaryotic protein of unknown function (DUF872)	3.6 e-13	2	233 nt	x	0	0
Pooc_B_c456	Serine/threonine-protein kinase CTR1	3.0 e-06	2	403 nt	x	0	0
Pooc_B_c46	no hit		2	420 nt	x	0	0
Pooc_B_c460	transition metal ion transport		2	827 nt	x	0	0
Pooc_B_c463	no hit		2	262 nt	x	0	0
Pooc_B_c464	Transcription factor e(y)2	4.9 e-08	2	423 nt	x	0	0
Pooc_B_c466	Photosystem I reaction center subunit V. chloroplastic	5.0 e-22	2	324 nt	x	0	0
Pooc_B_c467	no hit		2	252 nt	x	0	0
Pooc_B_c47	Chlorophyll a-b binding protein M9. chloroplastic	9.0 e-21	2	374 nt	x	0	0
Pooc_B_c470	transport		2	460 nt	x	0	0
Pooc_B_c476	no hit		2	264 nt	x	0	0
Pooc_B_c479	no hit		2	408 nt	x	0	0
Pooc_B_c48	3-ketoacyl-CoA synthase 6	3.0 e-27	2	258 nt	x	0	0
Pooc_B_c49	Zinc finger CCCH domain-containing protein 8	2.0 e-29	2	483 nt	x	0	0
Pooc_B_c52	60S ribosomal protein L19	2.0 e-09	2	340 nt	x	0	0
Pooc_B_c53	cytoplasmic vesicle		2	341 nt	x	0	0
Pooc_B_c55	Universal stress protein A-like protein	6.0 e-10	2	583 nt	x	0	0
Pooc_B_c56	no hit		2	206 nt	x	0	0

Pooc_B_c57	no hit		2	371 nt	x	0	0
Pooc_B_c59	catalytic activity		2	295 nt	x	0	0
Pooc_B_c6	no hit		2	407 nt	x	0	0
Pooc_B_c60	establishment of localization		2	317 nt	x	0	0
Pooc_B_c62	no hit		2	487 nt	x	0	0
Pooc_B_c63	40S ribosomal protein S3a	1.0 e-26	2	279 nt	x	0	0
Pooc_B_c64	Chlorophyll a-b binding protein 8. chloroplastic	1.0 e-06	2	192 nt	x	0	0
Pooc_B_c65	Glyceraldehyde-3-phosphate dehydrogenase A. chloroplastic	6.0 e-36	2	330 nt	x	0	0
Pooc_B_c66	no hit		2	470 nt	x	0	0
Pooc_B_c67	no hit		2	278 nt	x	0	0
Pooc_B_c74	Cyanate hydratase	0	2	668 nt	x	0	0
Pooc_B_c75	Pyridoxal biosynthesis protein PDX1	0	2	439 nt	x	0	0
Pooc_B_c77	Cytochrome P450 85A1	0	2	558 nt	x	0	0
Pooc_B_c78	Katanin p60 ATPase-containing subunit	0	2	621 nt	x	0	0
Pooc_B_c79	no hit		2	234 nt		0	0
Pooc_B_c82	Zinc finger A20 and AN1 domain-containing stress-associated protein 5	1.0 e-08	2	325 nt	x	1	0
Pooc_B_c86	no hit		2	305 nt	x	0	0
Pooc_B_c88	no hit		2	387 nt	x	0	0
Pooc_B_c90	Uncharacterized GMC-type oxidoreductase y4nJ	8.0 e-06	2	358 nt	x	0	0
Pooc_B_c92	no hit		2	236 nt	x	0	0
Pooc_B_c94	Photosystem I reaction center subunit VI-1. chloroplastic	2.0 e-32	2	318 nt	x	0	0
Pooc_B_c96	no hit		2	115 nt		0	0
Pooc_B_c8	cytoplasmic vesicle		1	449 nt	x	0	0
Pooc_B_c207	response to light stimulus		1	533 nt	x	0	0
Pooc_B_c201	Fructose-bisphosphate aldolase. chloroplastic	0	1	363 nt	x	0	0
Pooc_B_c307	Zeaxanthin epoxidase. chloroplastic	8.0 e-07	1	515 nt	x	0	0
Pooc_B_c418	metabolic process		1	546 nt	x	0	0
Pooc_B_c293	Chlorophyll a-b binding protein 1. chloroplastic	3.0 e-14	1	580 nt	x	0	0
Pooc_B_rp1_B2_R	no hit		1	290 nt	x	0	0
Pooc_B_rp1_B3_F	no hit		1	364 nt	x	0	0
Pooc_B_rp1_B4_F	Fasciclin-like arabinogalactan protein 16	8.0 e-17	1	315 nt	x	0	0
Pooc_B_rp1_C2_F	no hit		1	283 nt	x	0	0
Pooc_B_rp1_E8_R	no hit		1	169 nt	x	0	0
Pooc_B_rp1_F5_F	no hit		1	484 nt	x	0	0
Pooc_B_rp1_G3_R	no hit		1	336 nt	x	0	0
Pooc_B_rp1_H6_R	no hit		1	123 nt		0	0
Pooc_B_rp1_R12_R	Dual specificity protein phosphatase 12	4.0 e-14	1	566 nt	x	0	0
Pooc_B_rp10_A12_F	Chlorophyll a-b binding protein 151. chloroplastic	0	1	634 nt	x	0	0
Pooc_B_rp10_A8_F	no hit		1	445 nt	x	0	0
Pooc_B_rp10_C1_R	no hit		1	367 nt	x	0	0
Pooc_B_rp10_C6_R	no hit		1	973 nt	x	0	0
Pooc_B_rp10_D10_R	no hit		1	324 nt	x	0	0
Pooc_B_rp10_D12_R	no hit		1	178 nt	x	0	0

Pooc_B_rp10_D5_F	V-type proton ATPase 16 kDa proteolipid subunit	3.0 e-15	1	420 nt	x	0	0
Pooc_B_rp10_E10_R	Histone H4	1.0 e-39	1	556 nt	x	0	0
Pooc_B_rp10_E3_F	no hit		1	464 nt	x	0	0
Pooc_B_rp10_G10_F	Chlorophyll a-b binding protein 21	0	1	704 nt	x	0	0
Pooc_B_rp10_G11_R	S-acyltransferase TIP1	8.0 e-38	1	733 nt	x	1	0
Pooc_B_rp10_G2_R	no hit		1	196 nt	x	0	0
Pooc_B_rp10_G4_F/R	40S ribosomal protein S8	4.0 e-39	1	607 nt	x	0	0
Pooc_B_rp10_G5_F	no hit		1	234 nt	x	0	0
Pooc_B_rp10_H5_F	GDP-mannose 3.5-epimerase 1	2.94 e-39	1	452 nt	x	0	0
Pooc_B_rp10_H5_R	GDP-mannose 3.5-epimerase 1	9.81 e-40	1	457 nt	x	0	0
Pooc_B_rp10_R7_R	Aquaporin TIP2-1	9.0 e-27	1	589 nt	x	1	0
Pooc_B_rp2_B3_R	Fasciclin-like arabinogalactan protein 16	7.0 e-24	1	311 nt	x	0	0
Pooc_B_rp2_B8_R	no hit		1	393 nt	x	0	0
Pooc_B_rp2_C2_F	no hit		1	227 nt	x	0	0
Pooc_B_rp2_D10_F/R	no hit		1	154 nt	x	0	0
Pooc_B_rp2_D11_F	no hit		1	356 nt	x	0	0
Pooc_B_rp2_D2_F/R	no hit		1	771 nt	x	0	0
Pooc_B_rp2_D3_R	no hit		1	42 nt		0	0
Pooc_B_rp2_D7_F	60S ribosomal protein L34	3.0 e-08	1	156 nt	x	0	0
Pooc_B_rp2_D9_F	no hit		1	160 nt	x	0	0
Pooc_B_rp2_E11_R	no hit		1	329 nt	x	1	0
Pooc_B_rp2_F2_F	no hit		1	183 nt	x	0	0
Pooc_B_rp2_F4_F	no hit		1	152 nt	x	1	0
Pooc_B_rp2_F8_F	no hit		1	182 nt	x	0	0
Pooc_B_rp2_G7_F	no hit		1	208 nt		1	0
Pooc_B_rp2_H1_F	no hit		1	238 nt	x	0	0
Pooc_B_rp2_R4_R	no hit		1	135 nt	x	0	0
Pooc_B_rp3_A5_R	no hit		1	124 nt	x	0	0
Pooc_B_rp3_A7_R	no hit		1	275 nt	x	0	0
Pooc_B_rp3_C10_F/R	Glutathione S-transferase 11	2.0 e-08	1	220 nt	x	0	0
Pooc_B_rp3_C11_R	no hit		1	144 nt		0	0
Pooc_B_rp3_D10_F	Chlorophyll a-b binding protein 40. chloroplastic	2.0 e-30	1	406 nt	x	0	0
Pooc_B_rp3_D3_R	Aspartate aminotransferase. chloroplastic	3.0 e-18	1	381 nt	x	0	0
Pooc_B_rp3_D5_R	no hit		1	224 nt	x	0	0
Pooc_B_rp3_E6_R	B2 protein	8.0 e-19	1	449 nt	x	0	0
Pooc_B_rp3_F10_F	no hit		1	206 nt	x	0	0
Pooc_B_rp3_F12_F	no hit		1	319 nt	x	0	0
Pooc_B_rp3_G12_F	Universal stress protein A-like protein	1.0 e-09	1	566 nt	x	0	0
Pooc_B_rp3_G6_F	Coatomer subunit beta'-1	5.0 e-38	1	470 nt	x	0	0
Pooc_B_rp3_H10_R	Eukaryotic translation initiation factor 2 subunit 3	4.0 e-27	1	313 nt	x	0	0
Pooc_B_rp3_R12_R	no hit		1	339 nt	x	1	0

Pooc_B_rp3_R6_R	no hit		1	360 nt	x	0	0
Pooc_B_rp4_A11_R	no hit		1	104 nt		0	0
Pooc_B_rp4_A7_F	no hit		1	290 nt	x	0	0
Pooc_B_rp4_A9_F	no hit		1	261 nt	x	0	0
Pooc_B_rp4_C12_F	Universal stress protein A-like protein	5.0 e-31	1	522 nt	x	0	0
Pooc_B_rp4_C3_F	no hit		1	482 nt	x	0	0
Pooc_B_rp4_D11_R	no hit		1	288 nt	x	0	0
Pooc_B_rp4_E4_F	Chlorophyll a-b binding protein 40. chloroplastic	4.06 e-39	1	462 nt	x	0	0
Pooc_B_rp4_E6_R	no hit		1	397 nt	x	1	0
Pooc_B_rp4_E7_F/R	no hit		1	786 nt	x	0	0
Pooc_B_rp4_F11_F	Ferredoxin. chloroplastic	2.0 e-16	1	379 nt	x	0	0
Pooc_B_rp4_G10_F	Pectinesterase-2	1.0 e-06	1	356 nt	x	0	0
Pooc_B_rp4_G3_F	no hit		1	446 nt	x	0	0
Pooc_B_rp4_G5_R	Chlorophyll a-b binding protein M9. chloroplastic	1.0 e-19	1	409 nt	x	0	0
Pooc_B_rp4_G9_F	no hit		1	247 nt	x	0	0
Pooc_B_rp4_H12_R	Chlorophyll a-b binding protein 40 . chloroplastic	2.0 e-32	1	406 nt	x	0	0
Pooc_B_rp4_H7_R	no hit		1	448 nt	x	0	0
Pooc_B_rp4_H8_R	no hit		1	300 nt	x	0	0
Pooc_B_rp4_R10_R	Monosaccharide-sensing protein 2	3.0 e-06	1	381 nt	x	0	0
Pooc_B_rp5_A4_R	BI1-like protein	1.0 e-17	1	472 nt	x	0	0
Pooc_B_rp5_B7_F	no hit		1	838 nt	x	0	0
Pooc_B_rp5_B8_R	Chlorophyll a-b binding protein 13. chloroplastic	8.0 e-30	1	295 nt	x	0	0
Pooc_B_rp5_D4_R	no hit		1	133 nt		0	0
Pooc_B_rp5_E11_R	no hit		1	215 nt	x	0	0
Pooc_B_rp5_E9_R	Fructose-1.6-bisphosphatase. cytosolic	2.0 e-36	1	372 nt	x	0	0
Pooc_B_rp5_F10_F	no hit		1	155 nt	x	0	0
Pooc_B_rp5_G2_F/R	Primary amine oxidase	1.0 e-19	1	385 nt	x	0	0
Pooc_B_rp5_G5_F	no hit		1	167 nt	x	0	0
Pooc_B_rp5_H1_F	no hit		1	890 nt	x	1	0
Pooc_B_rp5_H1_R	no hit		1	763 nt	x	0	0
Pooc_B_rp6_A10_R	Subtilisin-like protease	1.0 e-11	1	432 nt	x	0	0
Pooc_B_rp6_A12_R	no hit		1	110 nt		0	0
Pooc_B_rp6_B11_R	no hit		1	574 nt	x	0	0
Pooc_B_rp6_B9_F	no hit		1	225 nt	x	0	0
Pooc_B_rp6_C9_R	no hit		1	160 nt	x	0	0
Pooc_B_rp6_D10_F	no hit		1	720 nt	x	0	0
Pooc_B_rp6_D3_R	Auxin response factor 24	4.0 e-14	1	340 nt	x	0	0
Pooc_B_rp6_D4_F/R	no hit		1	493 nt	x	0	0
Pooc_B_rp6_D9_F	no hit		1	351 nt	x	0	0
Pooc_B_rp6_E11_F	no hit		1	448 nt	x	0	0
Pooc_B_rp6_E9_F	no hit		1	401 nt	x	0	0
Pooc_B_rp6_G1_R	SKP1-like protein 1B	2.0 e-10	1	406 nt	x	0	0

Pooc_B_rp6_G4_R	no hit		1	205 nt	x	0	0
Pooc_B_rp6_G6_F/R	no hit		1	143 nt	x	0	0
Pooc_B_rp6_H11_R	no hit		1	112 nt		0	0
Pooc_B_rp6_H2_F	no hit		1	175 nt	x	0	0
Pooc_B_rp6_R7_R	no hit		1	136 nt		0	0
Pooc_B_rp7_B2_F	no hit		1	349 nt	x	0	0
Pooc_B_rp7_B7_F	no hit		1	259 nt	x	0	0
Pooc_B_rp7_C4_F	no hit		1	170 nt	x	0	0
Pooc_B_rp7_C5_R	Chlorophyll a-b binding protein 40. chloroplastic	2.0 e-36	1	466 nt	x	0	0
Pooc_B_rp7_D11_R	Protochlorophyllide reductase C. chloroplastic	0	1	560 nt	x	0	0
Pooc_B_rp7_D2_F	no hit		1	336 nt	x	0	0
Pooc_B_rp7_E2_R	SCAR-like protein 1	2.0 e-21	1	922 nt	x	1	0
Pooc_B_rp7_E6_F	no hit		1	138 nt	x	0	0
Pooc_B_rp7_G1_F	no hit		1	242 nt	x	0	0
Pooc_B_rp7_G12_F	no hit		1	229 nt	x	0	0
Pooc_B_rp7_G2_R	no hit		1	336 nt	x	0	0
Pooc_B_rp7_H3_R	Cytochrome P450 71D10	2.0 e-17	1	425 nt	x	0	0
Pooc_B_rp7_R2_R	Probable plastid-lipid-associated protein 8. chloroplastic	8.0 e-17	1	252 nt	x	0	0
Pooc_B_rp8_A5_F	no hit		1	633 nt	x	0	0
Pooc_B_rp8_A6_F/R	Bifunctional dihydroflavonol 4-reductase/flavanone 4-reductase	7.0 e-38	1	676 nt	x	0	0
Pooc_B_rp8_A8_R	no hit		1	202 nt	x	0	0
Pooc_B_rp8_A9_R	no hit		1	379 nt	x	0	0
Pooc_B_rp8_B1_R	no hit		1	152 nt	x	0	0
Pooc_B_rp8_B4_R	no hit		1	129 nt	x	0	0
Pooc_B_rp8_C2_F/R	S-acyltransferase TIP1	0	1	866 nt	x	0	0
Pooc_B_rp8_D2_F	no hit		1	1005 nt	x	0	0
Pooc_B_rp8_D2_R	no hit		1	1018 nt	x	0	0
Pooc_B_rp8_D3_F	no hit		1	980 nt	x	0	0
Pooc_B_rp8_D5_F/R	DNA polymerase I	2.0 e-14	1	981 nt	x	0	0
Pooc_B_rp8_D7_R	Elongation factor G. chloroplastic	1.0 e-11	1	265 nt	x	0	0
Pooc_B_rp8_E1_F	no hit		1	374 nt	x	0	0
Pooc_B_rp8_E11_F	Chlorophyll a-b binding protein M9. chloroplastic	1.0 e-20	1	475 nt	x	0	0
Pooc_B_rp8_E6_R	no hit		1	123 nt		0	0
Pooc_B_rp8_F12_F	Chlorophyll a-b binding protein 1B. chloroplastic	5.00 e-37	1	641 nt	x	0	0
Pooc_B_rp8_G3_F/R	no hit		1	180 nt	x	0	0
Pooc_B_rp8_H12_F	no hit		1	629 nt	x	0	0
Pooc_B_rp8_R12_R	Chlorophyll a-b binding protein 1B. chloroplastic	4.00 e-37	1	532 nt	x	0	0
Pooc_B_rp9_B11_R	N-acetyltransferase 9-like protein	5.0 e-22	1	675 nt	x	0	0
Pooc_B_rp9_B5_R	no hit		1	686 nt	x	0	0
Pooc_B_rp9_C4_F	no hit		1	139 nt	x	0	0
Pooc_B_rp9_D1_R	Probable WRKY transcription factor	4.0 e-06	1	399 nt	x	0	0

Pooc_B_rp9_D4_R	Probable serine/threonine-protein kinase WNK4	3.0 e-09	1	604 nt	x	0	0
Pooc_B_rp9_E10_F	no hit		1	304 nt	x	1	0
Pooc_B_rp9_G10_F	Oxygen-evolving enhancer protein 3-2	2.0 e-37	1	480 nt	x	0	0
Pooc_B_rp9_G3_F	no hit		1	484 nt	x	0	0
Pooc_B_rp9_G4_R	Cytochrome P450 74A1. chloroplastic	7.0 e-15	1	363 nt	x	0	0
Pooc_B_rp9_G5_F	no hit		1	127 nt		0	0
Pooc_B_rp9_R1_R	no hit		1	324 nt	x	0	0
Pooc_B_rp9_R2_R	no hit		1	225nt	x	0	0
