# Supplementary material for: Acclimation to different depths by the marine angiosperm Posidonia oceanica: transcriptomic and proteomic profiles
Source: Front Plant Sci. 2013 Jun 17;4:195. doi: 10.3389/fpls.2013.00195 (PMC3683636; doi:10.3389/fpls.2013.00195)
Supplement: Table S3a — List of peptides identified in the 1DE gel of proteins from shallow samples (S). List of peptides identified in the slices of 1DE gel of proteins from shallow samples (S), the protein attribution obtained with GPM and X!TANDEM sotfwares with the corresponding log(e) value, functional annotation obtained with TBLASTN search against Dr.Zompo database and corresponding E-value are shown. [file DataSheet4.PDF]

Supplemental Table 3aS. List of peptides identified in the 23 slices of 1DE gel of proteins from shallow samples (S), the protein attribution obtained by GPM and X!TANDEM software with the corresponding log (e) value, functional annotation obtained with TBLASTN search against Dr.Zompo database and corresponding E-value are shown.

**Samples**	**Protein Attribution**	**Log(e) (Protein)**	**Peptide Sequences**	**TBLASTN (Dr.zompo)**	**E-value (Drzompo)**	**Functional Annotation**
2S	LOC_Os02g32030.1 gpmDB [0/0E0] homo (16/16)	-1.8	STLTDSLVAAAGIIAQEVAGDVR	Zoma_C_c23365	0.000007	Elongation factor 2
4S	gi|38154492| gb|AY368907|-1 gpmDB [21/29] homo (3/3) protein	-6.7	DLVLLLFETALLTSGFSLEEPNTFGNR	Pooc_Contig353	1.00e-10	Heat shock cognate protein 80
4S	At3g03780.1 gpmDB [101/133] homo (2/2) protein	-3.4	ALGVETVPVLVGPVSYLLLSK	Zoma_C_c68945	0.0005	5-methyltetrahydropteroyltriglutamate—homocysteine methyltransferase
4S	gi|38154488| gb|AY368906|-1 gpmDB [20/28] protein	3.3	MAEAETFAFQAEINQLLSLIINTFYSNK	Zoma_C_c66842	2.00e-08	Heat shock protein 81-3
5S	LOC_Os01g19450.1gpmDB [0/0E0] homo (18/18) gi|255556390|ref|XP_002519229.1|	-4.1	SSLVSGLLTIGPR	Zoma_C_c60330	0.75	D-3-phosphoglycerate dehydrogenase
5S	LOC_Os04g55720.1:	-3	GLIEPISSVFVNLVNADFTAK	Zoma_C_c60244	0.0001	D-3-phosphoglycerate dehydrogenase. chloroplastic
5S	gi|32987735|gb|AK102526|1	-1.9	QDRPVSVQASQLHKSNNSR	No hits		
6S	gi|32978364| gb|AK068346|1 gpmDB [0/0E0] protein		QECGGGGGGGGGGGDENPR	No hits		
6S	gi|157980300| gb|EX528572|-3 gpmDB [9/15] homo (3/3)	-3.2	AGGPLGEVVDPLYPGGSFDPLGLAEDPEAFAELK	Pooc_B_c408	4.00e-16	Chlorophyll a-b binding protein 1B. chloroplastic

	protein					
6S	gi|157980300| gb|EX528572|-3 gpmDB [9/15] homo (5/5) protein		IAGGPLGEVVDPLYPGGSFDPLGLAEDPEAFAELK	Pooc_B_c408	1.00e-16	Chlorophyll a-b binding protein 1B. chloroplastic
6S	gi|28393479| gb|BT004140|-2 gpmDB [1/1] protein	-1.5	FDPILLGSQAASTNTGMQIQIESSDR	No hits		
7S	gi|147784163| gpmDB [0/6]	-79	IAQIPVSEAYLGR	Zoma_C_c64410	1.1	ATP synthase subunit alpha. chloroplastic
7S	gi|147784163| gpmDB [0/6]		LIESPAPGIISR	Zoma_C_c64410	1.7	ATP synthase subunit alpha. chloroplastic
7S	gi|147784163| gpmDB [0/6]		SVYEPLQTGLIAIDSMIPIGR	Zoma_C_c64410	0.00002	ATP synthase subunit alpha. chloroplastic
7S	gi|147784163| gpmDB [0/6]		ASSVAQVVTTFQER	Zoma_C_c64410	0.29	ATP synthase subunit alpha. chloroplastic
7S	gi|147784163| gpmDB [0/6]		EAYPGDVFYLHSR	Zoma_C_c59649	0.010	ATP synthase subunit alpha. chloroplastic
7S	(H) LOC_Os04g16740.1 gpmDB [0/0E0] homo (5/29) protein	-55.6	IAQIPVSEAYLGR	Zoma_C_c64410	1.1	ATP synthase subunit alpha. chloroplastic
7S	(H) LOC_Os04g16740.1 gpmDB [0/0E0] homo (5/29) protein		LIESPAPGIISR	Zoma_C_c64410	1.7	ATP synthase subunit alpha. chloroplastic
7S	(H) LOC_Os04g16740.1 gpmDB [0/0E0] homo (5/29) protein		SVYEPLQTGLIAIDSMIPIGR	Zoma_C_c64410	0.00001	ATP synthase subunit alpha. chloroplastic
7S	(H) LOC_Os04g16740.1 gpmDB [0/0E0] homo (5/29) protein		TAVATDTILNQK	Zoma_C_c64410	1.8	ATP synthase subunit alpha. chloroplastic
7S	(H) LOC_Os04g16740.1 gpmDB [0/0E0] homo (5/29) protein		EAYPGDVFYLHSR	Zoma_C_c59649	0.012	ATP synthase subunit alpha. chloroplastic

7S	sp|A6MMJ2|ATPA_DIOEL	-41.1	LIESPAPGIISR	Zoma_C_c64410	1.7	ATP synthase subunit alpha. chloroplastic
7S	sp|A6MMJ2|ATPA_DIOEL		SVYEPLQTGLIAIDSMIPIGR	Zoma_C_c64410	0.00001	ATP synthase subunit alpha. chloroplastic
7S	sp|A6MMJ2|ATPA_DIOEL		ASSVAQVVTTFQER	Zoma_C_c64410	0.29	ATP synthase subunit alpha. chloroplastic
7S	sp|A6MMJ2|ATPA_DIOEL		EAYPGDVFYLHSR	Zoma_C_c59649	0.012	ATP synthase subunit alpha. chloroplastic
7S	sp|A6MMJ2|ATPA_DIOEL		LELAQFAELEAFAQFASDLDK	Zoma_C_c59649	0.010	ATP synthase subunit alpha. chloroplastic
7S	ATCG00480.1 gpmDB [72/113] homo(6/19) protein	-32.5	IAQIIGPVLDVAFPPGK	Zoma_C_c22857	0.024	ATP synthase subunit beta. chloroplastic
7S	ATCG00480.1 gpmDB [72/113] homo(6/19) protein		TVLIMELINNIAK	Zoma_C_c42615	0.71	ATP synthase subunit beta. chloroplastic
7S	ATCG00480.1 gpmDB [72/113] homo(6/19) protein		DVNEQDVLLFIDNIFRFVQAGS EVSA	Zoma_C_c61380	0.001	ATP synthase subunit beta. chloroplastic
7S	ATCG00480.1 gpmDB [72/113] homo(6/19) protein		DVNEQDVLLFIDNIFR	Zoma_C_c61380	0.001	ATP synthase subunit beta. chloroplastic
7S	tr|Q4FGI4|Q4FGI4_TYPLA	-16.8	TVLIMELINNIAK	Zoma_C_c42615 Zoma_C_c61380	0.71	ATP synthase subunit beta. chloroplastic
7S	tr|Q4FGI4|Q4FGI4_TYPLA		DVNEQDVLLFIDNIFR	Zoma_C_c61380	0.001	ATP synthase subunit beta. chloroplastic
7S	gi|54651587| gb|BT016806|-2	-11.8	DAEGQDVLLFIDNIFR	Zoma_C_c22399	0.001	ATP synthase subunit beta. mitochondrial
7S	gi|54651587| gb|BT016806|-3		ITDEFTGAGAIGQVCQVIGAVV DVR	Zoma_C_c11144	6.00E-08	ATP synthase subunit beta. mitochondrial
7S	tr|F2DJJ2|F2DJJ2_HORVD	-10	DAEGQDVLLFIDNIFR	Zoma_C_c22399	0.001	ATP synthase subunit beta. mitochondrial
7S	tr|F2DJJ2|F2DJJ2_HORVD		ITDEFTGAGSVGQVCQVIGAV VDVR	Zoma_C:c11144	6.00E-08	ATP synthase subunit beta. mitochondrial
7S	gi|32966579| gb|AY328025|-3	-19.4	TFQGPPHGIQVER	Pooc_B_rp6_D3 _R	2.7	No Swiss-Prot Hits

7S	gi|32966579| gb|AY328025|-4		EMTLGFVDLLR	No hits		
7S	gi|32966579| gb|AY328025|-5		WSPELAAACEVWK	Pooc_B_rs2_H9_R	7.9	No Swiss-Prot Hits
7S	At2g28000.1 gpmDB [61/93] homo (5/5) protein	-10.2	GGYPILIIAEDIEQEALATLVVNK	Zoma_C_c58171	0.0000003	RuBisCO large subunit-binding protein subunit beta. chloroplastic
7S	At3g23810.1 gpmDB [83/146] homo (10/10) protein	-4.2	WVFPDTNSGIIVLAEGR	Zoma_C_c68954	0.001	Adenosylhomocysteinase 1
7S	gi|119350|sp|P25696.1	-2.8	SGETEDTFIADLAVGLSTGQIK	Zoma_C_c67987	0.000004	Enolase 1
7S	LOC_Os06g37180.1 gpmDB [0/0E0] homo (51/51) protein	-2.7	NIFQSLDLAWTLLR	Pooc_Contig181	2.2	V-type proton ATPase subunit B 2
8S	gi|210144177| gb|AK286959|-2gmpDB[0/13] homo (5/29) protein	-120	IAQIIGPVLDVAFPPGK	Zoma_C_c22857	0.024	ATP synthase subunit beta. chloroplastic
8S	gi|210144177| gb|AK286959|-2gmpDB[0/13] homo (5/29) protein		TVLIMELINNIAK	Zoma_C_c42615	0.71	ATP synthase subunit beta. mitochondrial
8S	gi|210144177| gb|AK286959|-2gmpDB[0/13] homo (5/29) protein		VGLTALTMAEYFR	Zoma_C_c22857	0.25	ATP synthase subunit beta. chloroplastic
8S	gi|210144177| gb|AK286959|-2gmpDB[0/13] homo (5/29) protein		DVNEQDVLLFIDNIFRFVQAGSEVSA	Zoma_C_c22857	8.00e-09	ATP synthase subunit beta. chloroplastic

8S	gi|210144177| gb|AK286959|-2gmp DB[0/13] homo (5/29) protein		DVNEQDVLLFIDNIFR	Zoma_C_c61380	0.001	ATP synthase subunit beta. chloroplastic
8S	gi|210144177| gb|AK286959|-2gmp DB[0/13] homo (5/29) protein		FVQAGSEVSALLGR	Zoma_C_c61380	0.22	ATP synthase subunit beta. chloroplastic
8S	gi|210144177| gb|AK286959|-2gmp DB[0/13] homo (5/29) protein		EGSITSIQAVYVPADDLTDP APATTFAHLDATTVLSR	Pooc_Contig181	0.097	V-type proton ATPase subunit B 2
8S	gi|210144177| gb|AK286959|-2gmp DB[0/13] homo (5/29) protein		YKELQDIIAILGLDELSEEDRLT VAR	Zoma_C_c61380	5.00e-08	ATP synthase subunit beta. chloroplastic
8S	gi|210144177| gb|AK286959|-2gmp DB[0/13] homo (5/29) protein		FLSQPFFVAEVFTGSPGKYVSL AETIRG	Zoma_C_c61380	5.00e-09	ATP synthase subunit beta. chloroplastic
8S	(H) gi|91983999| gpmDB [0/18] homo (6/29) protein		IAQIIGPVLDVAFPPGK	Zoma_C_c22857	0.024	ATP synthase subunit beta. chloroplastic
8S	(H) gi|91983999| gpmDB [0/18] homo (6/29) protein		IFNVLGEPVDNLGPVDTR	Zoma_C_c61380	0.0008	ATP synthase subunit beta. chloroplastic
8S	(H) gi|91983999| gpmDB [0/18] homo (6/29) protein	-112.6	TVLIMELINNIAK	Zoma_C_c42615	0.71	ATP synthase subunit beta. mitochondrial
8S	(H) gi|91983999| gpmDB [0/18] homo (6/29) protein		VGLTALTMAEYFR	Zoma_C_c22857	0.25	ATP synthase subunit beta. chloroplastic
8S	(H) gi|91983999| gpmDB		DVNEQDVLLFIDNIFRFVQAGS	Zoma_C_c22857	8.00e-09	ATP synthase subunit beta. chloroplastic

	[0/18] homo (6/29) protein		EVSA			
8S	(H) gi|91983999| gpmDB [0/18] homo (6/29) protein		DVNEQDVLLFIDNIFR	Zoma_C_c61380	0.001	ATP synthase subunit beta. chloroplastic
8S	(H) gi|91983999| gpmDB [0/18] homo (6/29) protein		FVQAGSEVSALLGR	Zoma_C_c61380	0.22	ATP synthase subunit beta. chloroplastic
8S	(H) gi|91983999| gpmDB [0/18] homo (6/29) protein		EGSITSIQAVYVPADDLTDPAPATTFAHLDATTVLSR	Pooc_Contig181	0.097	V-type proton ATPase subunit B 2
8S	(H) gi|91983999| gpmDB [0/18] homo (6/29) protein		YKELQDIIAILGLDELSEEDRLTVAR	Zoma_C_c61380	5.00e-08	ATP synthase subunit beta. chloroplastic
8S	(H) gi|91983999| gpmDB [0/18] homo (6/29) protein		ELQDIIAILGLDELSEEDR	Zoma_C_c61380	0.0009	ATP synthase subunit beta. chloroplastic
8S	ATCG00490.1 gpmDB [74/111] homo (0/33)protein	-56.9	YHIEPVPGEETQFIAYVAYPLDLFEEGSVTNM	Zoma_C_c22377	3.00e-11	Ribulose bisphosphate carboxylase large chain
8S	ATCG00490.1 gpmDB [74/111] homo (0/33)protein		VAYPLDLFEEGSVTNMFTSIVGNVFGFK	Ppc_B_c314	1.0	No swiss Prot Hits
8S	ATCG00490.1 gpmDB [74/111] homo (0/33)protein		TFQGPPHGIQVER	Pooc_B_rp6_D3_R	2.7	Auxin response factor 24
8S	ATCG00490.1 gpmDB [74/111] homo (0/33)protein		GGLDFTKDDENVNSQPFMR	Zoma_C_c22377	0.000006	Ribulose bisphosphate carboxylase large chain
8S	ATCG00490.1 gpmDB [74/111] homo (0/33)protein		FLFCAEAIYK	Zoma_C_c22377	1.9	Ribulose bisphosphate carboxylase large chain
8S	ATCG00490.1 gpmDB [74/111] homo (0/33)protein		WSPELAAACEVWK	Pooc_B_rs2_H9_R	7.9	No Swiss-Prot Hits
8S	(H) gi|91984000| gpmDB [1/16] homo (6/33) protein	-47.1	VAYPLDLFEEGSVTNMFTSIVGNVFGFK	Pooc_B_c314	1.0	No Swiss-Prot Hits
8S	(H) gi|91984000| gpmDB		TFQGPPHGIQVER	Zoma_C_c22377	0.012	Ribulose bisphosphate carboxylase large

	[1/16] homo (6/33) protein					chain
8S	(H) gi|91984000| gpmDB [1/16] homo (6/33) protein		GGLDFTKDDENVNSQPFMR	Zoma_C_c22377	0.000006	Ribulose bisphosphate carboxylase large chain
8S	(H) gi|91984000| gpmDB [1/16] homo (6/33) protein		EITLGFVDLLR	No hits		
8S	(H) gi|91984000| gpmDB [1/16] homo (6/33) protein		WSPELAAACEVWK	Zoma_C_c33689	0.013	Ribulose bisphosphate carboxylase large chain
8S	sp|P25414.1|RBL_AEGTA	-27.5	AVTAESSTGTWTTVWTDGLTSLDR	Pooc_B_c314	1.0	No Swiss-Prot Hits
8S	sp|P25414.1|RBL_AEGTA		VAYPLDLFEEGSVTNMFTSIVGNVFGFK	Pooc_B_c314	1.0	No Swiss-Prot Hits
8S	sp|P25414.1|RBL_AEGTA		TFQGPPHGIQVER	Zoma_C_c22377	0.012	Ribulose bisphosphate carboxylase large chain
8S	sp|P34767.1|RBL_ALIPL	-23.2	VSPQPGVPAEEAGAAVAAESSTGTWTTVWTDGLTSLDR	Pooc_B_c314	1.0	No Swiss-Prot Hits
8S	sp|P34767.1|RBL_ALIPL		VAYPLDLFEEGSVTNMFTSIVGNVFGFK	Pooc_B_c314	1.0	No Swiss-Prot Hits
8S	sp|P34767.1|RBL_ALIPL		TFQGPPHGIQVER	Zoma_C_c22377	0.012	Ribulose bisphosphate carboxylase large chain
9S	gi|12620882| gb|AF329935|-2 gpmDB [3/4] protein	-12.2	VPIIVTGNDFSTLYAPLIR	Pooc_Contig343	1.0	Ribulose bisphosphate carboxylase/oxygenase activase A. chloroplastic
9S			TDGVRDEDIVKLVDTFPGQSIDFFGALR	Pooc_Contig343	1.0	Ribulose bisphosphate carboxylase/oxygenase activase A. chloroplastic
9S	At4g20360.1 gpmDB [57/83] homo (1/1) protein	-1.5	QTELPFLLAVEDVFSITGR 167	Zoma_C_c37103	0.0001	Elongation factor TuB. chloroplastic

10S	gi|156725011| gb|EV229122|-1 gpmDB [1/7] homo (2/93) protein	-30.2	DDVELVAVNDPFITTDYMTYMFK	Pooc_Contig14 Pooc_Contig14	3.00e-09	Glyceraldehyde-3-phosphate dehydrogenase
10S	gi|156725011| gb|EV229122|-1 gpmDB [1/7] homo (2/93) protein		NPEEIPWGETGAEFVVESTGVFTDK		4.00e-10	Glyceraldehyde-3-phosphate dehydrogenase
10S	gi|156725011| gb|EV229122|-1 gpmDB [1/7] homo (2/93) protein		FGIVEGLMTTVHSITATQK	Pooc_Contig14	6e-05.	Glyceraldehyde-3-phosphate dehydrogenase
10S	gi|156725011| gb|EV229122|-1 gpmDB [1/7] homo (2/93) protein		VPTVDVSVVDLTVR	Pooc_Contig14	0.009	Glyceraldehyde-3-phosphate dehydrogenase
10S	gi|85720767| gb|DQ355800|-2 gpmDB [1/8] homo (1/25)	-29.5	DDVELVAVNDPFITTDYMTYMFK	Pooc_Contig14 Pooc_Contig14	3.00e-09	Glyceraldehyde-3-phosphate dehydrogenase
10S	gi|85720767| gb|DQ355800|-2 gpmDB [1/8] homo (1/25)		FGIVEGLMTTVHSITATQK		6.00e-05	Glyceraldehyde-3-phosphate dehydrogenase
10S	gi|85720767| gb|DQ355800|-2 gpmDB [1/8] homo (1/25)		VPTVDVSVVDLTVR	Pooc_Contig14	0.009	Glyceraldehyde-3-phosphate dehydrogenase
10S	gi|85720767| gb|DQ355800|-2 gpmDB [1/8] homo (1/25)		LVSWYDNEWGYSSR	Pooc_Contig14	0.00004	Glyceraldehyde-3-phosphate dehydrogenase
10S	At1g12900.1 gpmDB [57/84] homo (5/24) protein	-12.5	VPTPNVSVVDLVVQVSK	Pooc_B_c65	0.027	Glyceraldehyde-3-phosphate dehydrogenase
10S	At1g12900.1 gpmDB [57/84] homo (5/24) protein		VVDLADIVANNWKVVDLADIVAN	Pooc_Contig14	3.00e-09	Glyceraldehyde-3-phosphate dehydrogenase
10S	sp|P08477|G3PC_HORVU	-9.5	FGIVEGLMTTVHAMTATQK	Pooc_Contig14	0.00002	Glyceraldehyde-3-phosphate dehydrogenase
10S	sp|P08477|G3PC_HORVU		VPTVDVSVVDLTVR	Pooc_Contig14	0.009	Glyceraldehyde-3-phosphate

						dehydrogenase
10S	tr|A6YED2|A6YED2_LOLPR	-9.5	FGIVEGLMTTVHAMTATQK	Pooc_Contig14	0.00002	Glyceraldehyde-3-phosphate dehydrogenase
10S	tr|A6YED2|A6YED2_LOLPR		VPTVDVSVVDLTVR	Pooc_Contig14	0.009	Glyceraldehyde-3-phosphate dehydrogenase
10S	gi|82621107| gb|DQ284454|-3 gpmDB [8/10] homo (0/3) protein	-8.6	NDLEFAKKLASLADLYVNDAFGTAHR	Pooc_PC021E08 Zoma_C_c22889	2.00e-09	Phosphoglycerate kinase. cytosolic
10S	gi|82621107| gb|DQ284454|-3 gpmDB [8/10] homo (0/3) protein		IGVIESLLEKVDVLLLGGGMIFTFYK		9.00e-08	Phosphoglycerate kinase. chloroplastic
10S	gi|48752579| gb|CO083098|-3 gpmDB [10/11] homo (1/1) protein	-3.1	TLLVSAPGLGDYISGAILFEETLYQSTIDGK	Pooc_PC035C04	4.00e-12	Fructose-bisphosphate aldolase. chloroplastic
10S	gi|150162092| gb|EE553762|-1 gpmDB [0/1] protein	-2.4	DALFKHANIKPIITSTVWK	Pooc_PC044A11	0.22	Plant protein 1589 of unknown function
10S	Pooc_Contig14_2	-11.2	VPTVDVSVVDLTVR	Pooc_Contig14	0.009	Glyceraldehyde-3-phosphate dehydrogenase
10S	Pooc_Contig14_2		GILGYVEEDLVSTDFVGDCR	Pooc_Contig14	0.0000002	Glyceraldehyde-3-phosphate dehydrogenase
11S	Zoma_B_i02521_4	-18.3	LYSIASSAIGDFGDSK	Zoma_C_c7377	0.012	Ferredoxin--NADP reductase. leaf isozyme. chloroplastic
11S	Zoma_B_i02521_4		GLAWLFLGVPTSSSLLYK	Zoma_C_c53834	0.00007	Ferredoxin--NADP reductase. chloroplastic
11S	Zoma_B_i02521_4		MAEYAEELWELLK	Zoma_C_c22465	0.22	Ferredoxin--NADP reductase. leaf isozyme. chloroplastic
11S	gi|54651500| gb|BT016719|-2 gpmDB [1/3] homo (2/17)	-11.3	VAILGAAGGIGQPLSLLMK	Zoma_C_c49176	0.001	Malate dehydrogenase. mitochondrial

	protein					
11S	gi|54651500| gb|BT016719|-2 gpmDB [1/3] homo (2/17) protein		GLNGVPDIVECSFVQSTVTELPFFASK	Pooc_PC026E10	0.74	Cysteine proteinase RD19a
12S	sp|Q6ENG0|CYF_ORYNI	-9.5	YSEIVFPILSPDPAMK	Zoma_C_c42478 Zoma_C_c47693	0.014	Apocytochrome f
12S	sp|Q6ENG0|CYF_ORYNI		GPELLVSEGESIK		1.1	Apocytochrome f
12S	gi|73880486| gb|DT483224|3 gpmDB [0/1] homo (5/5) protein	-1.9	NPLNYTQVSVLADDILK	Zoma_C_c45955	0.004	ATP synthase subunit gamma. mitochondrial
13S	gi|156738236| gb|EV242357|2 gpmDB [0/8] homo (2/43) protein	-38.3	DGIDYAAVTVQLPGGER	Pooc_B_c195 Pooc_B_c296	1.00e-06	Oxygen-evolving enhancer protein 1. chloroplastic
13S	gi|156738236| gb|EV242357|2 gpmDB [0/8] homo (2/43) protein		SKPETGEVIGVFESIQPSDTDLGAK	Pooc_B_c296	8.00e-10	Oxygen-evolving enhancer protein 1. chloroplastic
13S	gi|156738236| gb|EV242357|2 gpmDB [0/8] homo (2/43) protein		GTGTANQCPTIDGGSESFPFK	Pooc_Contig378	3.00e-08	Oxygen-evolving enhancer protein 1. chloroplastic
13S	gi|19156| gb|Z11999|-1 gpmDB [6/15] protein	-35.2	DGIDYAAVTVQLPGGER	Pooc_B_c195	1.00e-06	Oxygen-evolving enhancer protein 1. chloroplastic
13S	gi|19156| gb|Z11999|-1 gpmDB [6/15] protein		GGSTGYDNAVALPAGGR	Pooc_B_c296	1.00e-13	Oxygen-evolving enhancer protein 1. chloroplastic
13S	gi|19156| gb|Z11999|-1 gpmDB [6/15] protein		ASLTGKITLSVTQSKPETGEVIGVFESIQPSDTDLGAK	Pooc_B_c296	1.00e-13	Oxygen-evolving enhancer protein 1. chloroplastic
13S	Pooc_Contig281_1	-22.7	CVEYEFLEETFGPK	Pooc_Contig281	0.0005	Probable glutathione S-transferase GSTU6
13S	Pooc_Contig281_1		AAFLGQLLEATQLLEGAFEK	Pooc_Contig281	3.00e-06	Probable glutathione S-transferase

						GSTU6
13S	Pooc_Contig281_1		ILDEDTTPLLLGWAER	Pooc_Contig281	0.00007	Probable glutathione S-transferase GSTU6
14S	gi|47953819| gb|CN825750|1 gpmDB [0/4] homo (2/2) protein	-15	LYPGGSFFDPLGLAADPEKK	Pooc_B_c132	0.0008	Chlorophyll a-b binding protein 21. chloroplastic
14S	Zoma_C_c28128_4	-9.7	WAMLGTLGCVFPELPSR	Pooc_B_rp10_G10_F	0.032	Chlorophyll a-b binding protein 21. chloroplastic
14S	gi|93179911| gb|CJ722764|-3 gpmDB [3/17] homo (1/5) protein	-9.2	VAGGPLGEIVDPLYPGGSFDPLGLAEDPEAFAELK	Pooc_B_rp8_F12_F	3.00e-15	Chlorophyll a-b binding protein 1B. chloroplastic
14S	At2g21170.1 gpmDB [43/65] homo (1/8) protein	-8.8	EEDIDGFLVGGASLK	Zoma_C_c10171	0.076	Triosephosphate isomerase. chloroplastic
14S	At2g21170.1 gpmDB [43/65] homo (1/8) protein		GPEFATIVNSVTSK	Zoma_C_c22367	0.47	Triosephosphate isomerase. chloroplastic
14S	gi|110373880| gb|EC938302|3 gpmDB [2/3] protein	-4.9	QEDIDGFLVGGASLKGPEFAVIVNSVT	Zoma_C_c22367	6.00e-09	Triosephosphate isomerase. chloroplastic
14S	LOC_Os07g37240.1 gpmDB [0/0E0] homo (3/3) protein	-3.4	STPFQPYTEVFGLQR	Pooc_B_c272	0.00001	Chlorophyll a-b binding protein CP29.1. chloroplastic
15S	gi|66738106| gb|DR015736|1 gpmDB [0/5] homo (9/51) protein	-15.8	GRPLGEVTDPIYPGGSFDPLGLADDPDAFA ELK	Pooc_B_c132	2.00e-12	Chlorophyll a-b binding protein 21. chloroplastic
15S	gi|66738106| gb|DR015736|1 gpmDB [0/5] homo (9/51) protein		GPIENLADHLADPVNNNAWAYATNFVPGK	Pooc_B_rs7_B11_F	2.00e-13	Chlorophyll a-b binding protein M9. chloroplastic
15S	Pooc_B_rp7_C5_R_6	-12.7	NSPNSFDPLGLAEDPEAFAELK	Pooc_B_rp7_C5_R	2.00e-08	Chlorophyll a-b binding protein 40. chloroplastic

15S	Pooc_B_rp7_C5_R_6		GPLENLADHLADPVNNNAWAYATNFVPGK	Pooc_B_rs7_B11_F	1.00e-13	Chlorophyll a-b binding protein M9. chloroplastic
15S	sp|P12330|CB21_ORYSJ	-12.2	LYLGPLSGEPPSYLTGEFPGDYGWDTAGLSADPETFAK	Pooc_B_c360	9.00e-16	Chlorophyll a-b binding protein M9. chloroplastic
15S	sp|P12330|CB21_ORYSJ		GPLENLADHLADPVNNNAWAYATNFVPGK	Pooc_B_rs7_B11_F	1.00e-13	Chlorophyll a-b binding protein M9. chloroplastic
15S	tr|B3G0G4|B3G0G4_9ASPA	-12	IAGGPLGEVTDPLYPGGSFDPLGLADDPEAFSELK	Pooc_B_c132	1.00e-14	Chlorophyll a-b binding protein 21. chloroplastic
15S	tr|B3G0G4|B3G0G4_9ASPA		GPLENLADHLADPVNNNAWAYATNFVPGK	Pooc_B_rs7_B11_F	1.00e-13	Chlorophyll a-b binding protein M9. chloroplastic
16S	gi|48389884| gb|CN917384|-3 gpmDB [9/10] protein	-2.9	NEVPVISPEQLAEADGIIFGFPTR	Zoma_C_c56810	0.0003	Flavoprotein wrbA
16S	gi|52390306| gb|CV233595|3 gpmDB [0/1] homo (12/12)	-2.7	LTGTDVGYPGGLWFDPLGWGSGSPEK	Pooc_B_c320	0.28	Chlorophyll a-b binding protein CP24 10A. chloroplastic
16S	gi|67029385|gb|CO458134|2: reversed protein	-2.3	HLDLSVVLAAPLRVVCLSAGR	No hits		
17S	Pooc_PC028C07_2	-19.6	VFFDMTIGAAPAGR	Pooc_PC028C07	0.001	Peptidyl-prolyl cis-trans isomerase
17S	Pooc_PC028C07_2		IVMELYADVVPR	Pooc_PC028C07	0.005	Peptidyl-prolyl cis-trans isomerase
17S	Pooc_PC028C07_2		HVVFGQIVDG IDVVR	Pooc_PC028C07	0.001	Peptidyl-prolyl cis-trans isomerase
17S	gi|73875282| gb|DT478020|-3 gpmDB [0/1] homo (2/2) protein	-6	IVIGLFGDDVPQTAENFR	Pooc_PC015E05	0.0008	Peptidyl-prolyl cis-trans isomerase CYP20-1
17S	gi|211854988| gb|DV989166|1 gpmDB [0/0E0] homo (2/2) protein	-3.5	HVVFGQVVEGLDVVR	Pooc_PC028C07	0.002	Peptidyl-prolyl cis-trans isomerase
17S	At1g07790.1 gpmDB [67/132] homo (232/232)	-3.2	AMGIMNSFINDIFEK	Zoma_C_c37957	0.008	Histone H2B

	protein					
17S	gi|73878969| gb|DT481707|-2 gpmDB [0/0E0] homo (11/11) protein	-3.1	VVFVPWVETDFR	Pooc_B_c478	0.26	No Swiss-Prot Hits
17S	gi|38605705|sp|P05642.2|	-3.1	IVTGVPEAIPVIGSPLVELLR	Zoma_C_c33944	0.00009	Cytochrome b6
18S	gi|83984616| gb|DW130725|-3 gpmDB [1/2] homo (2/25) protein	-10.2	QGLLCGSDGLPHLIVSGDQR	Pooc_B_c271	0.016	Photosystem I reaction center subunit III. chloroplastic
18S	gi|83984616| gb|DW130725|-3 gpmDB [1/2] homo (2/25) protein		GFIWPVAAYR	Pooc_B_c271	0.043	Photosystem I reaction center subunit III. chloroplastic
18S	At1g07790.1 gpmDB [67/132] homo (232/232) protein	-1.9	AMGIMNSFINDIFEK	Zoma_C_c37957	0.008	Histone H2B
21S	Pooc_Contig217_2	-3	AEEFGLLSLVEK	Pooc_Contig217	0.052	uncharacterized protein
22S	tr|A3A779|A3A779_ORYSJ	-2.2	RGPLASGAGAAALR	No hits		
22S	Pooc_Contig88_5	-2.1	EASGSVEVDDLVSDLK	Pooc_Contig88	0.0006	Uncharacterized protein At4g01150. chloroplastic
23S	ATCG01060.1 gpmDB [14/24] homo (5/5) protein	-2.6	IYDTCIGCTQCVR	Zoma_C_c36586	0.13	NAD(P)H-quinone oxidoreductase subunit H. chloroplastic
23S	ATCG01060.1 gpmDB [14/24] homo (5/5) protein		CESACPTDFLSVR	Zoma_C_c36586	0.14	NAD(P)H-quinone oxidoreductase subunit H. chloroplastic
