# Supplementary material for: Acclimation to different depths by the marine angiosperm Posidonia oceanica: transcriptomic and proteomic profiles
Source: Front Plant Sci. 2013 Jun 17;4:195. doi: 10.3389/fpls.2013.00195 (PMC3683636; doi:10.3389/fpls.2013.00195)
Supplement: Table S3b — List of peptides identified 1DE gel of proteins from deep samples (D). List of peptides identified in the slices of 1DE gel of proteins from deep samples (D), the protein attribution obtained with GPM and X!TANDEM softwares with the corresponding log(e) value, functional annotation obtained with TBLASTN search against Dr.Zompo database and corresponding E-value are shown. [file DataSheet5.PDF]

Supplemental Table 3bS. List of peptides identified in the 24 slices of 1DE gel of proteins from deep samples (D), the protein attribution obtained by GPM and X!TANDEM software with the corresponding log(e) value, functional annotation obtained with TBLASTN search against Dr.Zompo database and corresponding E-value are shown.

**SAMPLES**	**PROTEIN ATTRIBUTION**	**Log(e) protein**	**PEPTIDE SEQUENCES**	**TBLASTN (Dr.Zompo)**	**E-VALUE (Dr.Zompo)**	**FUNCTIONAL ANNOTATION**
1D	LOC_Os02g32030.1 gpmDB [0/0E0] homo (20/20)	-2.6	STLTDSLVAAAGIIAQEVAGDVR	Zoma_C_c23365	0.000007	Elongation factor 2
2D	LOC_Os02g32030.1 gpmDB [0/0E0] homo (20/20)	-1.4	STLTDSLVAAAGIIAQEVAGDVR	Zoma_C_c23365	0.000007	Elongation factor 2
3D	Pooc_PC044H12_2	-3.2	EVEDLEAAGINVIQIDEAALR	Pooc_PC044H12	0.000001	5-methyltetrahydropteroyltriglutamate
4D	sp|Q6L509|Q6L509_ORYSA	-11.2	IINEPTAAAIAYGLDK	Zoma_C_c66491	0.005	Heat shock cognate 70 kDa protein 2
5D	sp|Q93VT8.1|ACLB1_ORYSJ	-3.7	DLVSSLVSGLLTIGPR	Zoma_C_c60244	0.018	D-3-phosphoglycerate dehydrogenase , chloroplastic
6D	gi|194694909|gb|BT036534|-3	-1.8	GDVADGVFLGHADWPR	Pooc_B_c93	9.6	Probable eukaryotic translation initiation factor 5-2
6D	gi|116010686| gb|AK241321|-2	-1.8	GGECVGGGGGGGGGGAEAR	Pooc_Contig164	0.0000001	60S ribosomal protein L9
6D	gi|93228562| gb|CJ726612|-2	-1.6	TPFSSCGGAGAGAIPPPSR	Pooc_Contig176	0.84	60S acidic ribosomal protein P3
7D	gi|147784163| gpmDB [1/6] homo (0/28) protein	-68	IAQIPVSEAYLGR	Zoma_C_c64410	1.1	ATP synthase subunit alpha, chloroplastic
7D	gi|147784163| gpmDB [1/6] homo (0/28) protein		LIESPAPGIISR	Zoma_C_c59649	0.010	ATP synthase subunit alpha, chloroplastic
7D	gi|147784163| gpmDB [1/6] homo (0/28) protein		SVYEPLQTGLIAIDSMIPIGR	Zoma_C_c64410	0.00001	ATP synthase subunit alpha, chloroplastic
7D	gi|147784163| gpmDB [1/6]		ASSVAQVVTTFQER	Zoma_C_c64410	0.29	ATP synthase subunit alpha,

	homo (0/28) protein					chloroplastic
7D	gi|147784163| gpmDB [1/6] homo (0/28) protein		EAYPGDVFYLHSR	Zoma_C_c59649	0.012	ATP synthase subunit alpha, chloroplastic
7D	gi|147784163| gpmDB [1/6] homo (0/28) protein		LELAQFAELEAFAQFASDLDK	No hits		
7D	tr|H2F4C9|H2F4C9_9ASPA	-29.7	VVNTGTVLQVGDGIAR	Zoma_C_c22418	0.13	ATP synthase subunit alpha, chloroplastic
7D	tr|H2F4C9|H2F4C9_9ASPA		SVYEPLQTGLIAIDSMIPIGR	Zoma_C_c64410	0.00001	ATP synthase subunit alpha, chloroplastic
7D	tr|H2F4C9|H2F4C9_9ASPA		ASSVAQVVTTFQER	Zoma_C_c59649	0.010	ATP synthase subunit alpha, chloroplastic
7D	tr|H2F4C9|H2F4C9_9ASPA		EAYPGDVFYLHSR	Zoma_C_c59649	0.010	ATP synthase subunit alpha, chloroplastic
7D	tr|A9QBN6|A9QBN6_MUSAC	-19.1	SVYEPLQTGLIAIDSMIPIG	Zoma_C_c64410	0.00001	ATP synthase subunit alpha, chloroplastic
7D	tr|A9QBN6|A9QBN6_MUSAC		RASSVAQVVTTFRE	Zoma_C_c8917	2.2	ATP synthase subunit alpha, chloroplastic
7D	tr|A9QBN6|A9QBN6_MUSAC		EAYPGDVFYLHSR	Zoma_C_c59649	0.010	ATP synthase subunit alpha, chloroplastic
7D	gi|54651587| gb|BT016806|-2	-11.8	DAEGQDVLLFIDNIFR	Zoma_C_c22399	0.001	ATP synthase subunit beta, mitochondrial
7D	gi|54651587| gb|BT016806|-3		ITDEFTGAGAIGQVCQVIGAVVD VR	Zoma_C_c11144	6.00e-08	ATP synthase subunit beta, mitochondrial
7D	tr|F2DJJ2|F2DJJ2_HORVD	-10	DAEGQDVLLFIDNIFR	Zoma_C_c22399	0.001	ATP synthase subunit beta, mitochondrial
7D	tr|F2DJJ2|F2DJJ2_HORVD		ITDEFTGAGSVGQVCQVIGAVVD	Zoma_C_c11144	6.00e-08	ATP synthase subunit beta,

			VR			mitochondrial
7D	At2g28000.1 gpmDB [72/113] homo (6/19) protein	-9.3	APLLIIAEDVTGEALATLVVNK	Zoma_C_c57597	0.00003	RuBisCO large subunit-binding protein subunit alpha
8D	gi|210144177| gb|AK286959|-2 gpmDB [1/13] homo (0/29) protein	-93.3	IAQIIGPVLDVAFPPGK	Pooc_Contig181	0.097	V-type proton ATPase subunit B 2
8D	gi|210144177| gb|AK286959|-2 gpmDB [1/13] homo (0/29) protein		TVLIMELINNIAK	Zoma_C_c42615	0.71	ATP synthase subunit beta, mitochondrial
8D	gi|210144177| gb|AK286959|-2 gpmDB [1/13] homo (0/29) protein		DVNEQDVLLFIDNIFR	Zoma_C_c61380	0.001	ATP synthase subunit beta, chloroplastic
8D	gi|210144177| gb|AK286959|-2 gpmDB [1/13] homo (0/29) protein		FVQAGSEVSALLGR	Zoma_C_c61380	0.22	ATP synthase subunit beta, chloroplastic
8D	gi|210144177| gb|AK286959|-2 gpmDB [1/13] homo (0/29) protein		EGSITSIQAVYVPADDLTDPAPATTFAHLDATTVLSR	Pooc_Contig181	0.097	V-type proton ATPase subunit B 2
8D	gi|210144177| gb|AK286959|-2 gpmDB [1/13] homo (0/29) protein		YKELQDIIAILGLDELSEEDRLTVAR	Zoma_C_c61380	5.00e-08	ATP synthase subunit beta, chloroplastic
8D	gi|210144177| gb|AK286959|-2 gpmDB [1/13] homo (0/29) protein		ELQDIIAILGLDELSEEDR	Zoma_C_c61380	0.0009	ATP synthase subunit beta, chloroplastic
8D	gi|210144177| gb|AK286959|-2 gpmDB [1/13] homo (0/29) protein		FLSQPFFVAEVFTGSPGKYVSLAETIRG	Zoma_C_c61380	5.00e-09	ATP synthase subunit beta, chloroplastic
8D	(H) gi|91983999| gpmDB [0/18]	-83	IAQIIGPVLDVAFPPGK	Pooc_Contig181	0.097	V-type proton ATPase subunit B 2

	homo (6/29) protein					
8D	(H) gi|91983999| gpmDB [0/18] homo (6/29) protein		IFNVLGEPVDNLGPVDTR	Zoma_C_c61380	0.0008	ATP synthase subunit beta, chloroplastic
8D	(H) gi|91983999| gpmDB [0/18] homo (6/29) protein		TVLIMELINNIAK	Zoma_C_c42615	0.71	ATP synthase subunit beta, mitochondrial
8D	(H) gi|91983999| gpmDB [0/18] homo (6/29) protein		DVNEQDVLLFIDNIFR	Zoma_C_c61380	0.001	ATP synthase subunit beta, chloroplastic
8D	(H) gi|91983999| gpmDB [0/18] homo (6/29) protein		FVQAGSEVSALLGR	Zoma_C_c61380	0.22	ATP synthase subunit beta, chloroplastic
8D	(H) gi|91983999| gpmDB [0/18] homo (6/29) protein		EGSITSIQAVYVPADDLTDPAPATTFAHLDATTVLSR	Pooc_Contig181	0.097	V-type proton ATPase subunit B 2
8D	(H) gi|91983999| gpmDB [0/18] homo (6/29) protein		YKELQDIIAILGLDELSEEDRLTVAR	Zoma_C_c61380	5.00e-08	ATP synthase subunit beta, chloroplastic
8D	(H) gi|91983999| gpmDB [0/18] homo (6/29) protein		ELQDIIAILGLDELSEEDR	Zoma_C_c61380	0.0009	ATP synthase subunit beta, chloroplastic
8D	tr|Q4FGI4|Q4FGI4_TYPLA	-53.2	IAQIIGPVLDAVFPPGK	Pooc_B_c246	8.6	No Swiss-Prot Hits
8D	tr|Q4FGI4|Q4FGI4_TYPLA		IFNVLGEPVDNLGPVDTR	Zoma_C_c61380	0.0008	ATP synthase subunit beta, chloroplastic
8D	tr|Q4FGI4|Q4FGI4_TYPLA		TVLIMELINNIAK	Zoma_C_c42615	0.71	ATP synthase subunit beta, mitochondrial
8D	tr|Q4FGI4|Q4FGI4_TYPLA		DVNEQDVLLFIDNIFR	Zoma_C_c61380	0.001	ATP synthase subunit beta, chloroplastic
8D	tr|Q4FGI4|Q4FGI4_TYPLA		ELQDIIAILGLDELSEEDR	Zoma_C_c61380	0.0009	ATP synthase subunit beta, chloroplastic
8D	tr|H6THB1|H6THB1_9LILI	-52.7	IFNVLGEPVDNLGPVDTR	Zoma_C_c61380	0.0008	ATP synthase subunit beta, chloroplastic

8D	tr|H6THB1|H6THB1_9LILI		TVLIMELINNIAK	Zoma_C_c42615	0.71	ATP synthase subunit beta, mitochondrial
8D	tr|H6THB1|H6THB1_9LILI		DVNEQDVLLFIDNIFR	Zoma_C_c61380	0.001	ATP synthase subunit beta, chloroplastic
8D	tr|H6THB1|H6THB1_9LILI		ELQDIIAILGLDELSEEDR	Zoma_C_c61380	0.0009	ATP synthase subunit beta, chloroplastic
8D	tr|H2CPP4|H2CPP4_COLES	-52.6	IAQIIGPVLDVAFPPGK	Zoma_C_c22857	0.024	ATP synthase subunit beta, chloroplastic
8D	tr|H2CPP4|H2CPP4_COLES		TVLIMELINNIAK	Zoma_C_c42615	0.71	ATP synthase subunit beta, mitochondrial
8D	tr|H2CPP4|H2CPP4_COLES		DVNEQDVLLFIDNIFR	Zoma_C_c61380	0.001	ATP synthase subunit beta, chloroplastic
8D	tr|H2CPP4|H2CPP4_COLES		ELQDIIAILGLDELSEEDR	Zoma_C_c61380	0.0009	ATP synthase subunit beta, chloroplastic
8D	tr|H2CPP4|H2CPP4_COLES		LSQPFFVAEVFTGSPGKYVSLAETIRG	Pooc_B_c456	9.1	Serine/threonine-protein kinase CTR1
8D	tr|H6THB4|H6THB1_9LILI		IAQIIGPVLDVAFPPGK	Zoma_C_c22857	0.024	ATP synthase subunit beta, chloroplastic
8D	tr|H6THB4|H6THB1_9LILI		TVLIMELINNIAK	Zoma_C_c42615	0.71	ATP synthase subunit beta, chloroplastic
8D	tr|H6THB4|H6THB1_9LILI		DVNEQDVLLFIDNIFR	Zoma_C_c61380	0.001	ATP synthase subunit beta, chloroplastic
8D	tr|H6THB4|H6THB1_9LILI		ELQDIIAILGLDELSEEDR	Zoma_C_c61380	0.0009	ATP synthase subunit beta, chloroplastic
8D	tr|H6THB4|H6THB1_9LILI		LSQPFFVAEVFTGSPGKYVSLAETIRG	Zoma_C_c61380	2.00e-08	ATP synthase subunit beta, chloroplastic

8D	sp|Q85V44|ATPB_EUCBI	-50.1	IFNVLGEPVD NLGPVDTR	Zoma_C_c61380	0.0008	ATP synthase subunit beta, chloroplastic
8D	sp|Q85V44|ATPB_EUCBI		TVLIMELINNIAK	Zoma_C_c42615	0.71	ATP synthase subunit beta, mitochondrial
8D	sp|Q85V44|ATPB_EUCBI		DVNEQDVLLFIDNIFR	Zoma_C_c61380	0.001	ATP synthase subunit beta, chloroplastic
8D	sp|Q85V44|ATPB_EUCBI		ELQDIIAILGLDELSEEDR	Zoma_C_c61380	0.0009	ATP synthase subunit beta, chloroplastic
8D	sp|Q85V44|ATPB_EUCBI		FLSQPFFVAEVFTGSPGKYVGLTE TIRG	Zoma_C_c61380	5.00e-09	ATP synthase subunit beta, chloroplastic
8D	tr|H6THA9|H6THA9_9LILI	-49.6	IFNVLGEPVDNLGPVDTR	Zoma_C_c61380	0.0008	ATP synthase subunit beta, chloroplastic
8D	tr|H6THA9|H6THA9_9LILI		TVLIMELINNIAK	Zoma_C_c42615	0.71	ATP synthase subunit beta, mitochondrial
8D	tr|H6THA9|H6THA9_9LILI		DVNEQDVLLFIDNIFR	Zoma_C_c61380	0.001	ATP synthase subunit beta, chloroplastic
8D	tr|H6THA9|H6THA9_9LILI		ELQDIIAILGLDELSEEDR	Zoma_C_c61380	0.0009	ATP synthase subunit beta, chloroplastic
8D	tr|H6THA9|H6THA9_9LILI		GFQLILSGELDSLPEQAFYLVGNIE EATAK	Zoma_C_c61380	6.00e-11	ATP synthase subunit beta, chloroplastic
8D	gi|91984000| gpmDB [4/16] homo (10/23) protein	-20.7	VAYPLDLFEEGSVTNMFTSIVGN VFGFK	Zoma_C_c60354	8.00e-11	Ribulose bisphosphate carboxylase large chain
8D	gi|91984000| gpmDB [4/16] homo (10/23) protein		TFQGPPHGIQVER	Zoma_C_c22377	0.012	Ribulose bisphosphate carboxylase large chain
8D	gi|91984000| gpmDB [4/16] homo (10/23) protein		EITLGFVDLLR	No hits		

8D	(H) LOC_Os01g58020.1 gpmDB [0/0E0] homo (10/23) protein	-20.5	CYHIEPVVGEDNQYIAYVAYPLDLFEEGSVTNM	Pooc_B_c314		No Swiss-Prot Hits
8D	(H) LOC_Os01g58020.1 gpmDB [0/0E0] homo (10/23) protein		VAYPLDLFEEGSVTNMFTSIVGNVFGFK	Pooc_B_c314	1.0	No Swiss-Prot Hits
8D	(H) LOC_Os01g58020.1 gpmDB [0/0E0] homo (10/23) protein		TFQGPPHGIQVER	Zoma_C_c22377	0.012	Ribulose bisphosphate carboxylase large chain
8D	(H) ATCG00490.1 gpmDB [74/111] homo (10/23) protein	-19.8	VAYPLDLFEEGSVTNMFTSIVGNVFGFK	Zoma_C_c22377	1.00e-12	Ribulose bisphosphate carboxylase large chain
8D	(H) ATCG00490.1 gpmDB [74/111] homo (10/23) protein		TFQGPPHGIQVER	Zoma_C_c22377	0.012	Ribulose bisphosphate carboxylase large chain
8D	(H) ATCG00490.1 gpmDB [74/111] homo (10/23) protein		FLFCAEAIYK	Zoma_C_c22377	1.9	Ribulose bisphosphate carboxylase large chain
8D	tr|A1X8A0|A1X8A0_9POAL	-18.2	TFQGPPHGIQVER	Zoma_C_c22377	0.012	Ribulose bisphosphate carboxylase large chain
8D	tr|A1X8A0|A1X8A0_9POAL		FLFCAEALYK	Zoma_C_c22377	1.9	Ribulose bisphosphate carboxylase large chain
8D	tr|Q5IW67|Q5IW67_9ORYZ	-13	EGSITNMFTSIVGNVFGFK	Zoma_C_c60354	0.00004	Ribulose bisphosphate carboxylase large chain
8D	tr|Q5IW67|Q5IW67_9ORYZ		TFQGPPHGIQVER	Zoma_C_c22377	0.012	Ribulose bisphosphate carboxylase large chain
8D	tr|G8E9V4|G8E9V4_9POAL	-11.5	VAYPLDLFEEGSVTNMFTSNVGNVFGFK	Zoma_C_c60354	8.00e-10	Ribulose bisphosphate carboxylase large chain
8D	tr|G8E9V4|G8E9V4_9POAL		TFQGPPHGIQVER	Zoma_C_c22377	0.012	Ribulose bisphosphate carboxylase large chain

9D	gi|73873489| gb|DT476227|2 gpmDB [0/5] homo (0/29) protein	-16.6	VPIIVTGNDFSTLYAPLIR	Pooc_Contig343	1.0	Ribulose bisphosphate carboxylase/oxygenase activase A, chloroplastic
9D	gi|73873489| gb|DT476227|2 gpmDB [0/5] homo (0/29) protein		TDNVPEDDIVKLVDTFPGQSIDFF GALR	Pooc_Contig343	1.0	Ribulose bisphosphate carboxylase/oxygenase activase A, chloroplastic
9D	gi|12620882| gb|AF329935|-2 gpmDB [3/4] protein	-14.5	VPIIVTGNDFSTLYAPLIR	Pooc_Contig343	1.0	Ribulose bisphosphate carboxylase/oxygenase activase A, chloroplastic
9D	gi|12620882| gb|AF329935|-2 gpmDB [3/4] protein		TDGVRDEDIVKLVDTFPGQSIDFF GALR	Pooc_Contig343	1.0	Ribulose bisphosphate carboxylase/oxygenase activase A, chloroplastic
9D	LOC_Os04g56320.1	-13.8	IPLILGIWGGK	Pooc_Contig77	0.011	Ribulose bisphosphate carboxylase/oxygenase activase, chloroplastic
9D	LOC_Os04g56320.1		VPIIVTGNDFSTLYAPLIR	Zoma_C_c7563	0.00002	Ribulose bisphosphate carboxylase/oxygenase activase, chloroplastic
9D	gi|47104743| gb|BT013328|-1 gpmDB [14/22] homo (2/21) protein	-12.1	IGVIESLLEKVDVLLLGGGMIFTF YK	Pooc_PC037C07	0.054	Phosphoglycerate kinase, chloroplastic
9D	gi|47104743| gb|BT013328|-1 gpmDB [14/22] homo (2/21) protein		GVSLLLPTDVVIADK	Pooc_PC037C07	0.054	Phosphoglycerate kinase, chloroplastic
9D	gi|77540211| gb|DQ224371|-2 gpmDB [0/7] homo (1/12) protein	-11.5	VPTPNVSVVDLVVNVEK	No hits		
9D	gi|77540211| gb|DQ224371|-2 gpmDB [0/7] homo (1/12)		VVDLAHLVASK	No hits		

	protein					
9D	At4g20360.1 gpmDB [57/83] homo (1/1) protein	-2.5	QTELPFLLAVEDVFSITGR	Zoma_C_c37103	0.0001	Elongation factor TuB, chloroplastic
10D	gi|170240| gb|M14419|-1 pmDB2/6] homo (0/79) protein	-10.6	NPEEIPWAEAGADFVVESTGVFTDK	Zoma_C_c60400	7.00e-07	Glyceraldehyde-3-phosphate dehydrogenase
10D	gi|170240| gb|M14419|-1 pmDB2/6] homo (0/79) protein		VPTVDVSVVD	Pooc_Contig14	0.96	Glyceraldehyde-3-phosphate dehydrogenase
10D	gi|187950292| gb|AY103880|-3 gpmDB [0/1] protein	-4.8	LVDTNGAGDAFVGGFLSQLVLGK	Pooc_PC010D04	0.87	Fructokinase-2
10D	At5g66190.1 gpmDB [71/94] homo (3/30) protein	-24.1	LYSIASSAIGDFGDSK	Zoma_C_c7377	0.012	Ferredoxin--NADP reductase, leaf isozyme, chloroplastic
10D	At5g66190.1 gpmDB [71/94] homo (3/30) protein		GLAWLFLGVPTSSSLLYK	Zoma_C_c53834	0.00007	Ferredoxin--NADP reductase, chloroplastic
10D	At5g66190.1 gpmDB [71/94] homo (3/30) protein		MAEYAEELWELLK	Zoma_C_c22465	0.22	Ferredoxin--NADP reductase, leaf isozyme, chloroplastic
11D	gi|54651500| gb|BT016719|-2 gpmDB [1/3] homo (2/17) protein	-13.4	VAILGAAGGIGQPLSLLMK	Zoma_C-c49176	0.001	Malate dehydrogenase, mitochondrial
11D	gi|54651500| gb|BT016719|-2 gpmDB [1/3] homo (2/17) protein		GLNGVPDIVECSFVQSTVTELPFFASK	Pooc_PC026E10	0.74	Cysteine proteinase RD19a
11D	At5g66190.1gpmDB [71/94]homo (3/30) protein	-12.2	LYSIASSAIGDFGDSK	Zoma_C_c7377	0.012	Ferredoxin--NADP reductase, leaf isozyme, chloroplastic
11D	At5g66190.1gpmDB [71/94]homo (3/30) protein		MAEYAEELWELLK	Zoma_C_c22465	0.22	Ferredoxin--NADP reductase, leaf isozyme, chloroplastic
11D	Zoma_B_i02521_4	-10.5	LYSIASSAIGDFGDSK	Zoma_C_c7377	0.012	Ferredoxin--NADP reductase, leaf isozyme, chloroplastic

11D	Zoma_B_i02521_4		MAEYAEELWELLK	Zoma_C_c22465	0.22	Ferredoxin--NADP reductase, leaf isozyme, chloroplastic
11D	gi|76871077| gb|DV162069|-2:	-8.9	VAILGAAGGIGQPLSLLMK	Zoma_C_c49176	0.001	Malate dehydrogenase, mitochondrial
11D	gi|76871077| gb|DV162069|-2:		LNPLVSQLSLYDIAGTPGVA ADVSHINTR	Zoma_C_c22353	2.00e-09	Malate dehydrogenase, mitochondrial
12D	sp|Q6ENG0|CYF_ORYNI	-9.4	YSEIVFPILSPDPAMK	Zoma_C_c42478	0.014	Apocytochrome f
12D	sp|Q6ENG0|CYF_ORYNI		GPELLVSEGESIK	Zoma_C_c47693	1.1	Apocytochrome f
12D	gi|34959481| gb|CA106174|-2 gpmDB [0/1] protein	-1.7	AARPPPAGTPPPR	Pooc_PC019C02	0.26	Putative low molecular weight protein-tyrosine-phosphatase slr0328
13D	At3g50820.1 gpmDB [64/104] homo (6/9) protein	-12.7	YMEVKGTGTANQCPTIDGGSE	Pooc_B_c296	1.00e-09	Oxygen-evolving enhancer protein 1, chloroplastic
13D	At3g50820.1 gpmDB [64/104] homo (6/9) protein		SKPETGEVIGVFESLQPSDTDLGA K	Pooc_B_c296	1.00e-09	Oxygen-evolving enhancer protein 1, chloroplastic
13D	Pooc_Contig281_1	-12.1	CVEYEFLEETFGPK	Pooc_Contig281	0.0005	Probable glutathione S-transferase GSTU6
13D	Pooc_Contig281_1		AAFLGQLLEATQLLEGAFEK	Pooc_Contig281	0.000003	Probable glutathione S-transferase GSTU6
13D	gi|156738236|gb|EV242357|2 gpmDB [4/8] homo (1/33) protein	-9.4	SKPETGEVIGVFESIQPSDTDLGA K	Pooc_B_c296	8.00e-10	Oxygen-evolving enhancer protein 1, chloroplastic
13D	gi|19156| gb|Z11999|-1 gpmDB [6/15] protein	-9.6	DGIDYAAVTVQLPGGER	Pooc_B_c195	1.00e-06	Oxygen-evolving enhancer protein 1, chloroplastic
13D	gi|19156| gb|Z11999|-1 gpmDB [6/15] protein		ASSKGKITLSVTQSKPETGEVIGV FESIQPSDTDLGAK	Pooc_B_c296	1.00e-13	Oxygen-evolving enhancer protein 1, chloroplastic
13D	At5g65430.1gpmDB [53/81]homo (38/38) protein	-3.7	QAFEEAIAELDTLGEESYK	Pooc_PC039D11	0.00001	14-3-3-like protein C

14D	gi|47953819| gb|CN825750|1 gpmDB [1/4] homo (2/2) protein	-14.6	LYPGGSFFDPLGLAADPEKK	Pooc_B_c132	0.0008	Chlorophyll a-b binding protein 21, chloroplastic
14D	gi|110373880|gb|EC938302|3 g pmDB [0/3] homo (0/7) protein	-14	QEDIDGFLVGGASLK	Zoma_C_c59451	0.028	Triosephosphate isomerase, chloroplastic
14D	Zoma_C_c33371_3	-11.2	ELEVIHXRWAMLGTLGCVFPELL SR	Pooc_B_c360	0.0006	Chlorophyll a-b binding protein of LHCII type I, chloroplastic
14D	Zoma_C_c33371_3		WAMLGTLGCVFPELLSR 250	Pooc_Contig92	0.000007	Chlorophyll a-b binding protein 1B, chloroplastic
14D	LOC_Os07g37240.1 gpmDB [0/0E0] homo (3/3)	-4.8	STPFQPYTEVFGLQR	Pooc_B_c272	0.00001	Chlorophyll a-b binding protein CP29.1, chloroplastic
14D	At4g10340.1gpmDB [40/73]homo (12/12) protein	-4.1	TGALLLDGNTLNYFGK	Pooc_Contig159	0.00005	Chlorophyll a-b binding protein CP26, chloroplastic
15D	gi|157980300| gb|EX528572|-3 gpmDB [0/12] homo (0/15) protein	-29.5	YLGSFSGEAPSYLTGEFPGDYGW DTAGLSADPETFAK	Pooc_B_c360	1.00e-15	Chlorophyll a-b binding protein, chloroplastic
15D	gi|157980300| gb|EX528572|-3 gpmDB [0/12] homo (0/15) protein		WAMLGALGCVFPELLSR	Pooc_B_rp10_G10_F	0.018	Chlorophylla-b binding protein 21, chloroplastic
15D	gi|157980300| gb|EX528572|-3 gpmDB [0/12] homo (0/15) protein		IAGGPLGEVVDPLYPGGSFDPLGL AEDPEAFAELK	Pooc_B_rp8_F12_F	1.00e-15	Chlorophyll a-b binding protein 1B, chloroplastic
15D	gi|45990591| gb|CN149099|-3gpmDB [0/5]homo (9/51) protein	-12	TDEFPGDYGWDTAGLSADPETFA K	Pooc_B_c360	1.00e-09	Chlorophyll a-b binding protein of LHCII type I,
15D	gi|45990591| gb|CN149099|-3gpmDB [0/5]homo (9/51)		GPLENLADHLADPVNNNAWAYA TNFVPGK	Pooc_B_rs7_B11_F	1.00e-13	Chlorophyll a-b binding protein M9, chloroplastic

	protein					
15D	gi|73873524| gb|DT476262|2 gpmDB [0/6] homo (0/14) protein	-10.8	SEIPEYLTGEVPGDYGYDPFGLSK	Pooc_B_c360	0.00004	Chlorophyll a-b binding protein of LHCII type I, chloroplastic
15D	gi|73873524| gb|DT476262|2 gpmDB [0/6] homo (0/14) protein		TGALLLDGNTLNYFGK	Pooc_Contig159	5.00e-05	Chlorophyll a-b binding protein CP26, chloroplastic
15D	Zoma_C_c34383_5	-10.7	EPNSIFGVGGITMRRNTVK	Pooc_B_c132	0.005	Chlorophyll a-b binding protein 21, chloroplastic
15D	Zoma_C_c34383_5		WAMLGTLGCVFPELLSR	Pooc_Contig92	0.000007	Chlorophyll a-b binding protein 1B, chloroplastic
15D	Zoma_C_c15686_5	-10	ELEVIHTRWAMLGTLGCVFPELLSR	Pooc_B_c360	0.0006	Chlorophyll a-b binding protein of LHCII type I, chloroplastic
15D	Zoma_C_c15686_5		WAMLGTLGCVFPELLSR	Pooc_Contig92	0.000007	Chlorophyll a-b binding protein 1B, chloroplastic
15D	gi|35046182| gb|CA146793|-1gpmDB [0/3]homo (1/31)	-8.6	VIACVGETLEQR	Pooc_Contig109	0.015	Triosephosphate isomerase, cytosolic
15D	gi|35046182| gb|CA146793|-1gpmDB [0/3]homo (1/31)		NDWSNVVIAYEPVWAIGTGK	Pooc_Contig109	0.000009	Triosephosphate isomerase, cytosolic
16D	Pooc_Contig333_3	-21.8	SKVEDGIFGTSGGIGFTK	Pooc_Contig333	0.00003	Photosystem II 22 kDa protein, chloroplastic
16D	Pooc_Contig333_3		VEDGIFGTSGGIGFTK	No hits		
16D	Pooc_Contig333_3		VAMLGFAASIFGEAITGK	Pooc_Contig333	0.00003	Photosystem II 22 kDa protein, chloroplastic
17D	Pooc_PC028C07_2	-12.6	HVVFGQIVDGIDVVR	Pooc_PC028C07	1.2	No Swiss-Prot Hits
17D	Pooc_PC028C07_2		IVMELYADVVPR	Pooc_B_rp2_D2_R	0.002	Peptidyl-prolyl cis-trans isomerase

17D	gi|211854988|gb|DV989166|1 gpmDB	-2.1	HVVFGQVVEGLDVVR	Pooc_PC028C07	0.002	Peptidyl-prolyl cis-trans isomerase
18D	gi|83984616| gb|DW130725|-3 gpmDB [0/0E0] homo (2/25)	-21	QGLLCGSDGLPHLIVSGDQR	Pooc_Contig327	0.097	Photosystem I reaction center subunit III, chloroplastic
18D	gi|83984616| gb|DW130725|-3 gpmDB [0/0E0] homo (2/25)		GFIWPVAAYR	Pooc_B_c271	0.043	Photosystem I reaction center subunit III, chloroplastic
18D	Pooc_Contig132_3	-18	VWDFCASCQLMQLLPK	Pooc_Contig132	7.00e-07	S-norcoclaurine synthase
18D	Pooc_Contig132_3		TEIEGDGGVGTTTK	Pooc_PC011B10	0.001	14 kDa proline-rich protein DC2.15
18D	Pooc_Contig132_3		GTIEYEIGEGSSADPSLITTHSFATLSEAIAK	Pooc_B_rp9_G3_F	2.00e-10	No Swiss-Prot Hits
18D	Pooc_Contig48_2	-11.5	VWDFCGSSQLMQLLPK	Pooc_Contig48	0.000002	S-norcoclaurine synthase
18D	Pooc_Contig48_2		QIEGGHLDLGFLSSHSR	Pooc_Contig48	0.000007	S-norcoclaurine synthase
18D	At1g07790.1 gpmDB [67/132] homo (232/232) protein	-15.3	AMGIMNSFINDIFEK	Zoma_C_c37957	0.008	Histone H2B
19D	gi|37991269|gb|AK121646|3 gpmDB [0/2] protein	-1.9	IDTDQLMGIMGKPKTR	No hits		
19D	gi|7565762|gb|AW691026|1	-1.5	MEFGESGNMK	No hits		
21D	At5g38430.1 gpmDB [31/51] homo (6/6) protein	-3	EHGNTPGYYDGR	Pooc_Contig3	0.001	Ribulose bisphosphate carboxylase small chain SSU5B, chloroplastic
22D	LOC_Os01g61920.1 homo (267/267)	-2.5	TVTAMDVVYALK	Pooc_B_rp10_E10_R	0.018	Histone H4
22D	Pooc_Contig88_5	-2.4	EASGSVEVDDLVSDLK	Pooc_Contig88	0.0006	Uncharacterized protein At4g01150, chloroplastic
23D	ATCG01060.1 gpmDB [14/24] homo(10/13) protein	-11.8	IYDTCIGCTQCVR	Zoma_C_c36586	0.13	NAD(P)H-quinone oxidoreductase subunit H, chloroplastic

23D	ATCG01060.1 gpmDB [14/24] homo(10/13) protein		CESACPTDFLSVR	Zoma_C_c36586	0.14	NAD(P)H-quinone oxidoreductase subunit H, chloroplastic
23D	sp|Q3V4Y3|PSAC_ACOCL	-3.6	CESACPTDFLSVR	Zoma_C_c36586	0.14	NAD(P)H-quinone oxidoreductase subunit H, chloroplastic
23D	gi|27548338| gb|CA766549|	-3.3	FDSLEQLDEFSR	Zoma_C_c60643	1.6	Cytochrome b559 subunit alpha

5
